# Wireless Electrochemical Reactor for Accelerated Exploratory Study of Electroorganic Synthesis

**DOI:** 10.1021/acscentsci.3c00856

**Published:** 2023-09-05

**Authors:** Jie Chen, Yiming Mo

**Affiliations:** † College of Chemical and Biological Engineering, Zhejiang University, Hangzhou, 310027, Zhejiang, China; ‡ ZJU-Hangzhou Global Scientific and Technological Innovation Center, Zhejiang University, Hangzhou, 311215, Zhejiang, China

## Abstract

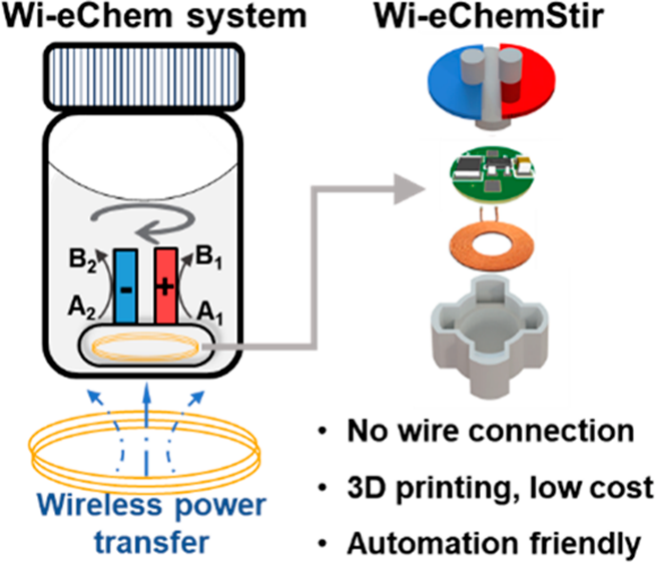

Electrosynthesis is an emerging tool to construct value-added fine chemicals under mild and sustainable conditions. However, the complex apparatus required impedes the facile development of new electrochemistry in the laboratory. Herein, we proposed and demonstrated the concept of wireless electrochemistry (Wi-eChem) based on wireless power transfer technology. The core of this concept is the dual-function wireless electrochemical magnetic stirrer that provides an electrolysis driving force and mixing simultaneously in a miniaturized form factor. This Wi-eChem system allowed electrochemists to execute electrochemical reactions in a manner similar to traditional organic chemistry without handling wire connections. The controllability, reusability, and versatility were validated with a series of modern electrosynthesis reactions, including electrodecarboxylative etherification, electroreductive olefin–ketone coupling, and electrochemical nickel-catalyzed oxygen atom transfer reaction. Its remarkably simplified operation enabled its facile integration into a fully automated robotic synthesis platform to achieve autonomous parallel electrosynthesis screening.

## Abstract 2

The wire-free electrosynthesis is realized with wireless power transfer technology, significantly reducing the operation complexity of executing the electrochemical reaction screening in the laboratory. This wireless electrosynthesis technology is automation friendly, providing a new route to automate electrosynthesis screening.

## Introduction

With the increasing demand for sustainable synthetic chemistry, electrosynthesis has recently gained the attention of synthetic chemists owing to its innate advantages in mild reaction conditions, use of traceless electrons as redox agents, and precise control of redox potentials.^1,2^ The wide potential range and unique heterogeneous electron transfer mechanism in the electrochemical system enables a number of useful transformations that, otherwise, would be challenging or impossible to accomplish using conventional methods.^3−5^ Up to now, the emergence of various electrosynthetic methods, such as electrochemical C–H functionalization^3,6^ and C–C,^7^ C–N,^8^ and C–S^9^ bond formations, offers alternative green and sustainable synthetic routes to the value-added fine chemicals or pharmaceuticals.

Despite its prominent advantages, electroorganic chemistry is still at the early discovery stage with limited practical applications. In addition to the complexity introduced by special apparatus^10^ and heterogeneous electron transfer,^11^ the parameters unique to electrosynthesis, including supporting electrolytes, electrode materials, and electrolysis current/voltage, significantly increase the reaction parametric space.^12^ Therefore, the optimization of electrochemical reaction conditions requires significantly more effort compared to conventional chemistry. To expedite the electrosynthesis discovery, both batch-type^13,14^ and flow-type^15−17^ electrochemical screening devices have been developed to increase experimental throughput and reduce operation complexity. The batch-type electrochemical cell is versatile and can adopt various electrode forms and multiphase reactions. Several standardized batch-type electrochemical devices, such as IKA ElectraSyn Carousel^12^ and HTe^–^Chem,^13^ have been commercialized to reduce the barrier of conducting parallelized reaction screening. In the flow-type electrosynthesis screening, microfluidic channels integrated with electrodes can intensify the interelectrode mass transfer and reduce the solution resistance, thus achieving an accelerated reaction rate.^18^

However, further streamlining the electrosynthesis development in the laboratory is still limited by the following challenges: (1) Recently developed electrosynthetic methods often involve radicals or transition metal catalysts, resulting in the oxygen- and/or moisture-sensitive nature of these reaction systems. The wires required for electrical connections cause tedious and error-prone cell sealing, leading to unreproducible or fluctuating reaction outcomes. (2) Automated high-throughput experimentation technology enables rapid reaction condition screening and optimization to streamline the development of organic chemistry. Nevertheless, the complex electrochemical cell structure and electrode preparation procedure makes it difficult to interface electrosynthesis with existing automated synthesis platforms. (3) Flow-type screening strategy is easy to automate, but it is limited to liquid or gas–liquid electrochemical reactions and only allows one reaction at a time, impeding its wide adoption in routine development of electrosynthesis.

Considering the remaining challenges above, the ultimate hope of practitioners in electrosynthesis is “Can electrochemical screening be executed just like the normal organic reaction performed in a glass vial with a magnetic stir bar?” Along with this goal, wire-free electricity transmission technology is a promising avenue to simplify the tedious and error-prone electrode connections required in electrosynthesis. Bipolar electrochemistry is one kind of wireless electrochemistry method, which utilizes the voltage applied on two feeder electrodes to promote electrochemical redox reactions at a bipolar electrode placed in electrolyte.^19,20^ The electrochemical driving force is transmitted through polarization of a bipolar electrode in the applied electric field. It has recently received extensive development in the field of sensors^21^ and material synthesis.^22^ However, the high voltage at the feeder electrodes to generate sufficient electric field required by the bipolar electrode may cause undesired electrochemical transformations, thus limiting its use in electrosynthesis, where the reaction selectivity is the top priority. In addition, near-field inductive power transfer is an efficient approach to drive electrical devices. Burek and co-workers implemented this technology to replace the conventional external irradiation with internal wireless LED illumination. The floating wireless LED bulbs driven by external alternating electromagnetic field significantly intensified the photon flux, especially when scaling-up the reactor.^23^

**Figure 1:**
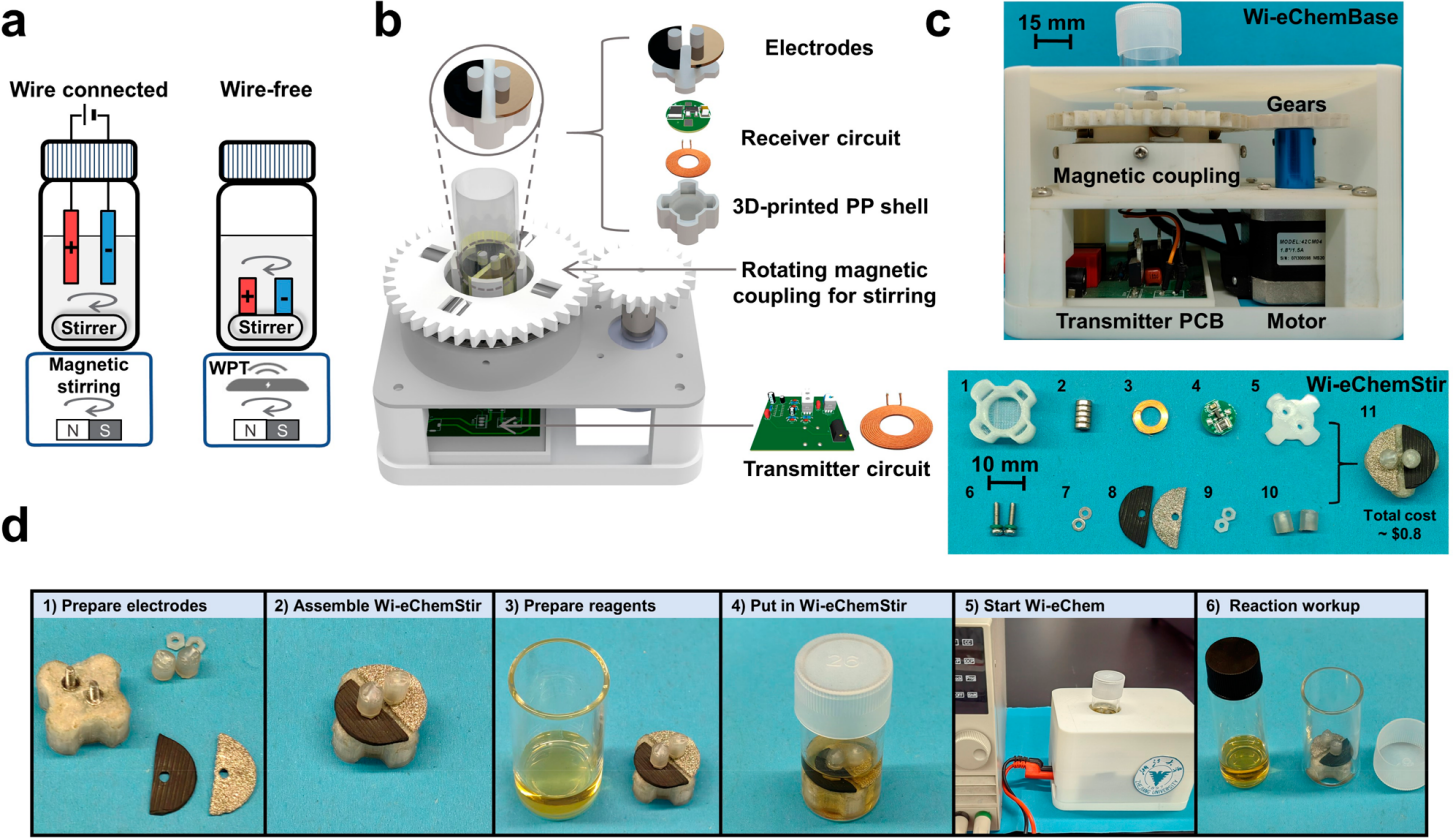
(a) Comparison between traditional electrochemical reactor and wireless electrochemical reactor. (b) CAD drawing of Wi-eChem system. (c) Wi-eChemBase and components inside Wi-eChemStir (1: 3D-printed PP shell, 2: magnets, 3: receiver coil, 4: receiver PCB, 5: PP lid, 6–7: 316L SS current collectors, 8: electrodes, 9: PP nuts, 10: PP caps). (d) The operation procedure of the Wi-eChem system.

In this work, we report the design of a wireless electrochemical system (Wi-eChem, Figure 1a,b) to streamline the exploration and optimization of electrosynthetic reactions. Wi-eChem relies on wireless power transfer (WPT) technology to achieve connection-free electrolysis. This design allows synthetic electrochemists to execute electrochemical reactions in a manner similar to traditional organic reaction by just putting the wireless electrochemical stir bar (Wi-eChemStir) inside a common reaction glass vial. Owing to its remarkably simplified electrosynthesis operation, a fully automated robotic electrosynthesis platform was constructed with a Wi-eChem system as the key enabling component to achieve autonomous parallel electrosynthesis screening.

## Results and Discussion

### Design of Wi-eChem System

The fundamental consideration for a Wi-eChem system is that it needs to be low-cost, easily accessible, and versatile for common electrosynthesis reactions, such that it could be widely adopted by electrosynthetic practitioners. Thus, we used 3D printing to construct all mechanical components for low-cost prototyping and technology transferability^24−26^ and a printed circuit board (PCB) for miniaturizing electrical connections.

First, we set out to engineer the geometric configuration of the Wi-eChem system. The core design is the use of two miniaturized inductively coupled coils to enable near-field wireless electricity transfer, which removes the requirement for physical wire connections to apply potential on electrodes. The transmitter coil (10 mm diameter) is located in an outer base (Wi-eChemBase), and the receiver coil (5 mm diameter) and its corresponding receiver PCB are embedded in a miniaturized in-vial electrochemical stirrer (Wi-eChemStir) (Figure 1b,c). Since the vial bottom is occupied by the wireless electricity transmission circuits, we engineered a ring-shaped magnetic coupling structure for driving Wi-eChemSitr to rotate inside the glass vial to introduce required mixing for reaction. Material selection for the Wi-eChemStir’s shell is the key to protecting the inner electronics from chemical corrosion. Among 3D-printable materials, easily accessible and chemical-resistant polypropylene (PP) appears to an ideal option.^24,25^ The final design of Wi-eChemStir has a dimension of 20 mm which can be fitted into a common glass vial, allowing a minimum reaction volume of 4 mL. The anode and cathode (18 mm semicircle shape) are attached to two 316L stainless steel (316L SS) current collectors, and the exposed 316L SS current collector is masked with a PP cap to avoid direct contact with electrolyte. For electrochemical reactions sensitive to the current distribution on the electrode, a modified Wi-eChemStir design with parallel electrodes was also constructed and demonstrated (see SI Section 3 and Section 8).

**Figure 2:**
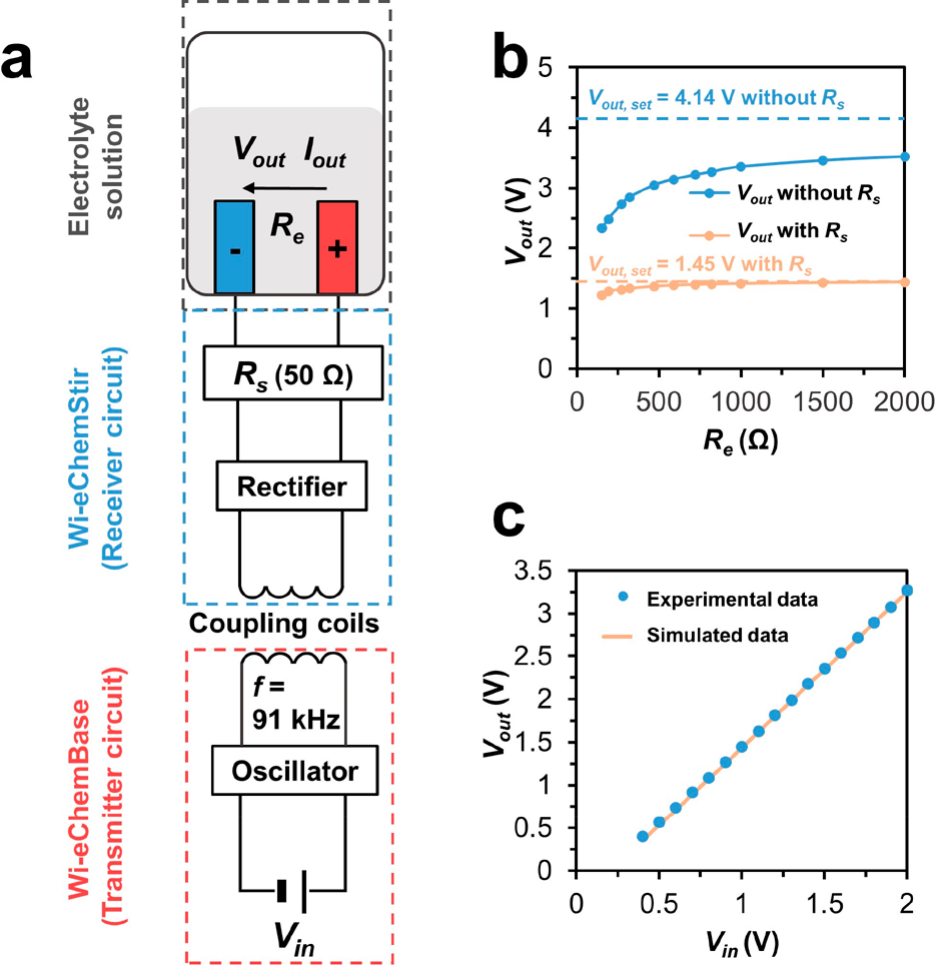
(a) The transmitter and receiver circuits of the Wi-eChem system. (b) The relationship of electrolysis voltage (*V*_out_) and equivalent resistance (*R*_e_) with and without stabilization resistor (*R*_s_) when *V*_in_ = 1.0 V. (c) Relationship of *V*_out_ and transmitter circuit input voltage (*V*_in_) obtained by MATLAB Simulink simulation and the experimental circuit measurement.

Next, we focused on engineering the electronics for the WPT to provide a sufficient, controllable, and stable electrifying potential to drive electrosynthesis. The wireless-transfer circuits (Figure 2a) modeled in MATLAB Simulink helped determine the specifications of each component (see SI Section 2). A 91 kHz high-frequency oscillating electromagnetic field was used for driving electrolysis to avoid the interference caused by a rotating magnetic field designated for mixing (∼10 Hz). As the equivalent electrolysis resistance (*R*_e_) varies with the performed electrochemical reactions and the progression of the electrolysis, electrolysis voltage (*V*_out_) could significantly deviate from the set value (Figure 2b). Thus, a stabilization resistor (*R*_s_) was configured in the receiver circuit to achieve a stable *V*_out_ over a typical range of *R*_e_ encountered in electrosynthesis (Figure 2a,b and Figure S4). In this way, *V*_out_ can be simply controlled according to the precalibrated relationship between *V*_out_ and the input voltage of the transmitter circuit (*V*_in_) (Figure 2c).

The final Wi-eChem system design was integrated into a compact chassis with a footprint of 134 mm × 94 mm × 75 mm (length × width × height), which can be easily multiplexed in the fume hood or automated synthesis platform. To operate the Wi-eChem system, synthetic electrochemists only need to put the Wi-eChemStir equipped with electrodes into the glass vial filled with reagents, and set the electrolysis voltage to initiate the electrochemical screening (Figure 1d). This procedure is similar to conducting traditional thermal chemistry, thus significantly simplifying the procedure for performing electrosynthesis (Video S1).

### Electrochemical Validation of Wi-eChem System

Based on this engineered Wi-eChem system, we validated its applicability in driving synthetic electrochemistry through two electrochemical visualization experiments.

**Figure 3:**
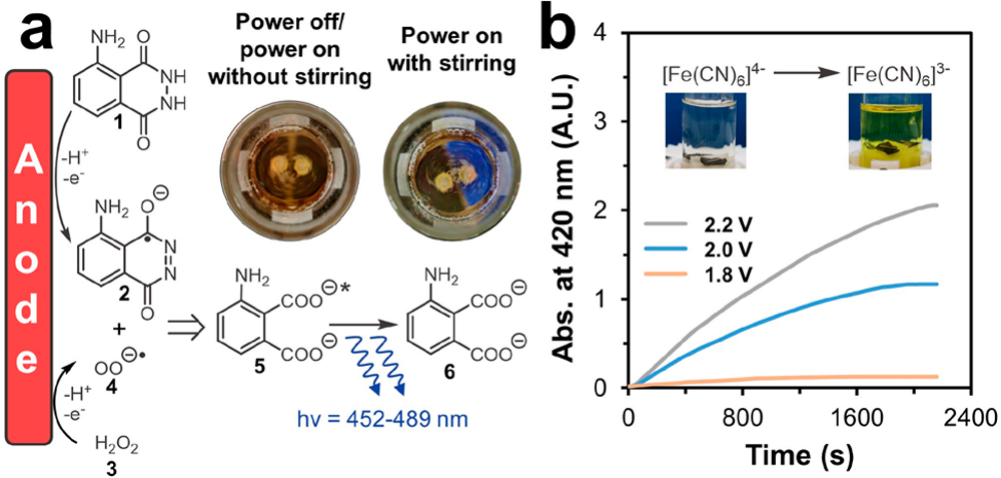
(a) The mechanism and experimental phenomena of luminol electrochemiluminescence reaction in the Wi-eChem system. (b) The absorbance at 420 nm for electrochemical oxidation of ferrocyanide to ferricyanide at different voltages.

First, we set out to demonstrate the electrolyzing and mixing dual function of Wi-eChem with the electrochemiluminescence (ECL) of luminol. Anodic oxidation and deprotonation transform the luminol (**1**) into its corresponding radical anion intermediate (**2**). It further reacts with superoxide radical (**4**) generated by hydrogen peroxide (**3**) oxidation to form the excited 3-aminophthalate (**5**), which emits blue light (452–489 nm).^27^ The lumino ECL offers a tool to visualize the oxidation events happening on the electrode surface. In the Wi-eChem system, the carbon paper anode surface illuminates blue light upon applying 3.0 V voltage (Figure 3a). When the Wi-eChemStir stopped rotating, the light emission gradually diminished because of the insufficient mass transfer of reagents to the electrode surface, indicating that reaction was limited by diffusion rate when without mechanical mixing. This validating example demonstrated that dual-function Wi-eChemStir provides good mass transfer and electrolysis driving force at the same time.

Adjusting the electrolysis voltage according to the electrosynthesis reaction performed is the fundamental requirement for an electrochemical device. The electrochemical oxidation of ferrocyanide to ferricyanide turns the originally colorless electrolyte solution into yellow, which can be spectroscopically monitored at 420 nm wavelength. The applied potential determines the reaction kinetics, where higher *V*_out_ should promote faster conversion. To monitor the progression of ferrocyanide oxidation, we used a peristaltic pump to circulate the electrolyte solution through an inline UV–Vis flow cell to track absorbance during electrolysis. As expected, the applied electrolysis potential directly affected the oxidation rate, which was reflected in the absorbance curve change rate and the final equilibrium concentration plateau (Figure 3b).

### Electrodecarboxylative Etherification

**Figure 4:**
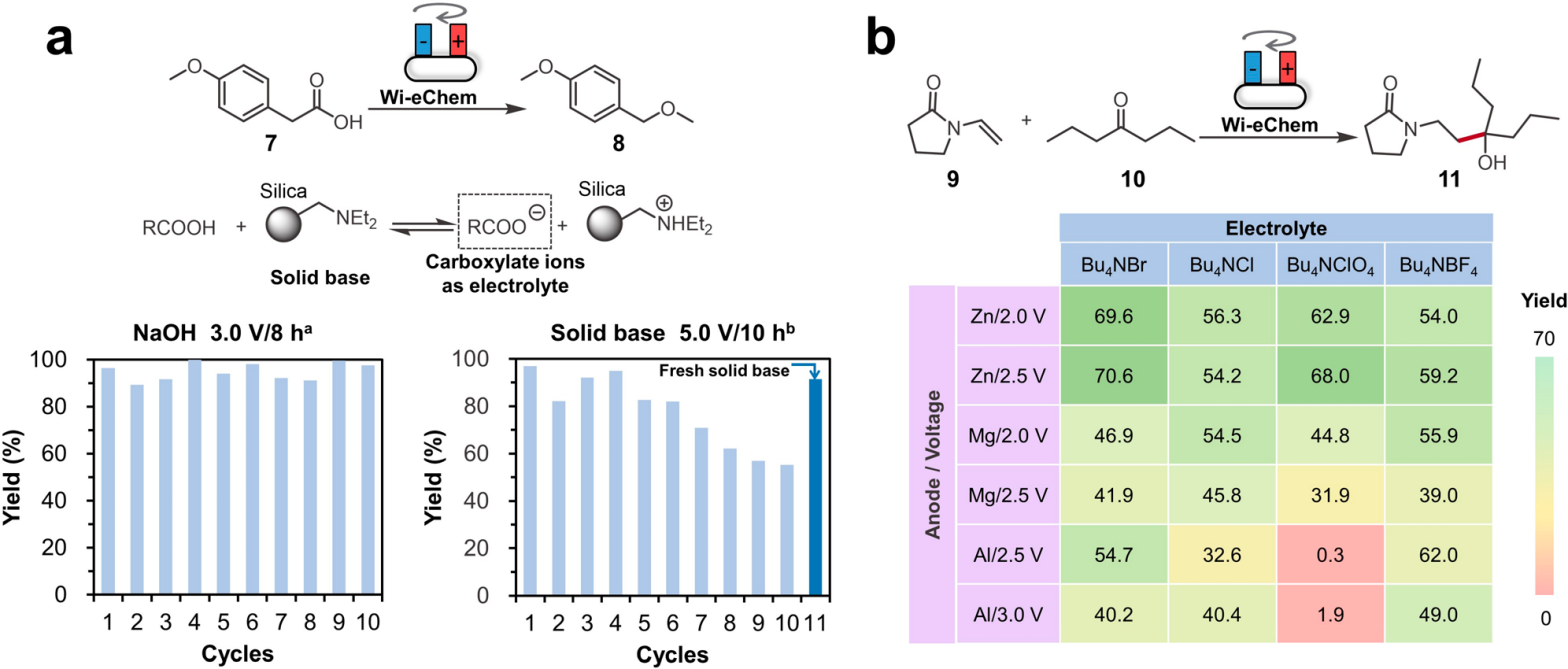
(a) Hofer-Moest electrodecarboxylative etherification using NaOH and a silica-supported solid base. Reaction conditions: ^a^**7** (0.4 mmol), NaOH (0.4 mmol), MeOH (4 mL), 3.0 V voltage, and 8 h duration with graphite (+)/nickel foam (−) as electrodes; ^b^**7** (0.4 mmol), solid base (0.4 g), MeOH (4 mL), 5.0 V voltage, and 10 h duration with graphite (+)/nickel sheet (−) as electrodes. (b) The screening results for various sacrificial anode materials, supporting electrolytes, and applied potentials in the electroreductive olefin-ketone coupling. **9** (1.4 mmol), **10** (2.8 mmol), electrolyte (1.2 mmol), and 16 h duration with Sn sheet as cathode. Yield for the two reactions above was determined by gas chromatography with trimethylbenzene as the internal standard.

With the confirmed capability of driving electrosynthesis, the durability and reusability of the designed Wi-eChemStir should also be verified despite its low cost (∼US$0.8 each). High-potential anodic reactions typically pose stringent requirements on electrodes and corresponding devices due to oxidative corrosion. We selected Hofer-Moest electrodecarboxylative etherification,^28^ an old electrochemical carbocation chemistry that has recently been re-exploited for its unique ability to synthesize challenging ethers,^29,30^ to assess the Wi-eChem system (Figure 4a). Ten repeated experiments (3.0 V and 8 h electrolysis) conducted with the same Wi-eChemStir gave stable yields (90–100%), and its service life exceeded 80 h without observing performance degradation, validating its reproducibility and reusability. Executing this reaction under a higher electrolysis potential (3.5 V) accelerated the reaction, thus reaching full conversion at a reduced reaction time of 3 h in 90% yield. Interestingly, it was found that the rotation speed of Wi-eChemStir played an important role in reaction selectivity control (Figure S18). A higher rotation speed induced an improved mass transfer rate for species renewal on the electrode surface, thus suppressing the overoxidation of the product (see SI Section 5 for detailed study of mass transfer effects).^31^

Furthermore, replacing the soluble NaOH with a solid base allows this reaction to proceed under the electrolyte-free condition,^32^ such that the downstream product purification is significantly simplified and the solid base can be recycled for reuse. We synthesized the silica-supported diethylamine solid base using a reported procedure (see SI Section 8 for detailed procedure).^33^ The carboxylate ions, derived from the acid–base reaction of the carboxylic acid and silica-supported base, behaved as self-supplied electrolytes. Due to the reduced solution conductivity, this reaction required up to 5.0 V to achieve a reasonable reaction rate, posing an even harsher condition on Wi-eChemStir compared to the soluble base scenario. Each time after the reaction, the silica-supported base was recycled by simple filtration for the next run. The first four cycles gave a good reaction yield, followed by a downward trend in the yield. This degraded performance was caused by the gradual loss of the anchored amine on silica, which was confirmed by elemental analysis (Table S3). Using a fresh silica-supported base with the used Wi-eChemStir restored the desired yield. After the intensive reusability tests above, the electronics inside the Wi-eChemSitr remained intact when opening the protection PP shell, further validating its chemical resistance and electronic stability.

### Electroreductive Olefin–Ketone Coupling

Now, the Wi-eChem system is ready for screening of parameters in the electroorganic reaction. We demonstrated Wi-eChem with a recently developed electrochemical C–C bond formation reaction, electroreductive olefin–ketone coupling.^7^ This reductive radical chemistry involves an ECEC mechanism. The ketone undergoes a single-electron reduction to generate the corresponding ketyl radical, which then goes through olefin trapping, a second single-electron reduction, and protonation to give the targeted C–C coupled tertiary alcohol. With Wi-eChemStir, we rapidly explored the combinations of three sacrificial anode materials (Zn, Mg, and Al), four supporting electrolytes (tetrabutylammonium electrolytes with different anions), and electrolysis voltages. Based on the screening results, the seemingly simple sacrificial anodic metal dissolving process and inert supporting electrolyte had significant impacts on the coupling efficiency. This is probably caused by the coordination of the electrochemically generated metal salts and reaction intermediates that affects the reduction thermodynamics.^34^ The combination of Bu_4_NClO_4_ and the aluminum anode completely failed to generate the desired product since the generated insoluble Al(ClO_4_)_3_ salt passivated the anode surface, thus preventing further metal dissolution (Figures S22–S23). Among other condition combinations, the reaction solution using the zinc electrode was relatively clear, and its yield was relatively high. The applied voltage determined the rate of the reaction, while it had minimum impact on the final yield. The highest yield was obtained by using zinc as the sacrificial anode and Bu_4_NBr as the electrolyte.

### Automated Electrosynthesis Screening Platform

**Figure 5:**
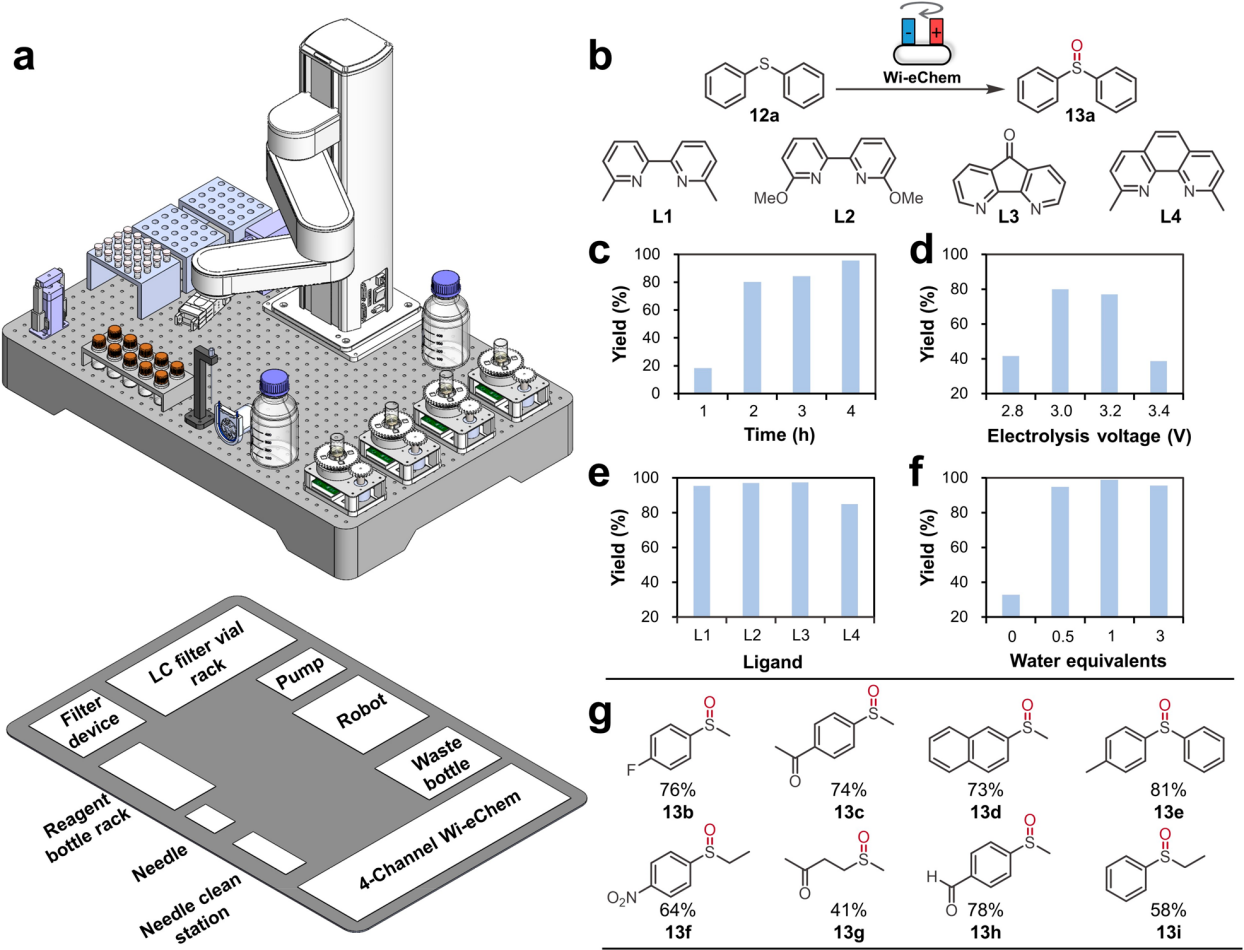
(a) CAD drawing of automated robotic electrosynthesis platform with four Wi-eChem systems with the projected floorplan indicating the function of each module. (b–g) Automated parameter investigation of electrochemical nickel-catalyzed oxygen atom transfer (OTA) oxygenation of organic sulfides. The influence of the reaction time (c), electrolysis voltage (d), ligands (e), water equivalents (f), and substrate type (g) was automatically investigated. Reaction condition: **12a** (0.2 mmol), NiCl_2_ (5 mol %), ligand (6 mol %), H_2_O (3 equiv except for Figure 5f) under 3.0 V voltage (except for Figure 4d) and 4 h duration (except for Figure 5c and 2 h duration for Figure 5d) with graphite (+)/nickel foam (−) as electrodes. Yield was determined by HPLC with naphthalene as the internal standard (Figure 5c–f) or ^1^H NMR with dibromomethane as the internal standard (Figure 5g).

Automated reaction screening and optimization has undergone extensive development in the past decade to accelerate the molecule discovery and process optimization.^35−38^ Especially, the recent emergence of modern artificial intelligence (AI) demands large datasets to train an accurate prediction neural model. Compared to thermal chemistry and photochemistry, complications caused by electrode insertion, wire connection, reactor sealing, and high-cost potentiostats impede the development of automated electrosynthesis platform.^39^ The Wi-eChem system developed in this work allows conducting electrochemistry with significantly reduced operation complexity, enabling its facile integration into a common automated synthesis workflow. Thus, we constructed a fully automated robotic electrosynthesis platform with four-channel parallelized Wi-eChem devices (Figure 5a).

On the hardware level, this platform is composed of five core functional modules: (1) A central SCARA robotic arm is responsible for physical connections of all modules, including physical movement of vials and needle. (2) A four-channel Wi-eChem system allows parallel high-throughput electrosynthesis screening. (3) The sample filtration module removes particulates from the reaction crude before liquid chromatography (LC) analysis. (4) The needle washing station cleans the liquid transfer needle and corresponding tubing to avoid cross-contamination. (5) The solution storage area keeps reagents and waste solutions.

On the software control level, we developed a “scheduler–worker” control scheme using LabVIEW as the graphical front-end and Python as the execution backend to maximize the automated experiment efficiency (see SI Section 10). This control scheme utilizes a central scheduler to allocate tasks to idle workers to execute a “preparation-reaction-workup-cleaning” experiment. Each worker waits in a first-come, first-served (FIFS) queue for each hardware module to complete each subtask. Therefore, the platform is flexible to accommodate parallel tasks with different subtask durations at a maximal module occupation ratio. To run automated electrosynthesis reactions, the user needs to prepare only an Excel file containing screening parameter information, such as reaction time, electrolysis voltage, and reagent volume.

To demonstrate the capability of this automated four-channel Wi-eChem platform, we considered an electrochemical nickel-catalyzed oxygen atom transfer (OTA) reaction.^40^ Mechanistically, the Ni(II)-bipyridine complex is electrochemically reduced to the Ni(I) intermediate, with subsequent activation of O_2_ from electrolytic water splitting to afford the Ni(II)-superoxo species, which then transfers an oxygen atom to the organic sulfide to yield sulfoxide. The influence of the electrolysis voltage, reaction time, ligands, water equivalents, and substrate structures was investigated (Figure 5b–g). We initiated the automated screening on the robotic Wi-eChem platform after preparing the reagent stock solutions and the experiment excel sheet (see Video S2). This automated platform rapidly identified that 3.0 V and 4 h was the optimal electrolysis condition. This reaction is relatively insensitive to choices of ligands. Water equivalence is an important factor in the occurrence of the reaction, which is in line with the mechanism that the reaction oxygen is derived from electrolyzed water. Substrate scope study under the optimal conditions was also performed with this automated platform to expand the substrate structure applicability (Figure 5g).

## Conclusion

In summary, the demonstrated wireless electrosynthesis technology allows synthetic electrochemists to perform electrochemical reactions with a much reduced effort. This Wi-eChem system can provide a sufficient and controllable driving force for common electrochemical reactions on the scale of milliliters. The chemical-resistant material selection and sealing techniques enable the reusability of Wi-eChemStir even under harsh chemical and electrochemical conditions. The open-sourced low-cost 3D-printed structural components and PCB electronics promote its adoption and further development. Most importantly, this design concept opens a new route to automating electrochemistry screening. Even though the current design has no feedback on electrolysis current, this can be realized using wireless communication technology (e.g., Bluetooth). Following this proof-of-concept work, we envision that additional advanced microelectronics can be integrated into such a wireless magnetic stirrer to enable feedback control and achieve real-time reaction monitoring using process analytical technology (PAT).

## Supporting Information Available

All experimental procedures, analysis, compound characterization data, CAD designs, and automation platform design (PDF)
Video for the usage of Wi-eChem system (MP4)
Video for the automated Wi-eChem screening platform (MP4)
Transparent Peer Review report available (PDF)
