# Supplementary material for: Wireless Electrochemical Reactor for Accelerated Exploratory Study of Electroorganic Synthesis
Source: ACS Cent Sci. 2023 Sep 5;9(9):1820–6. doi: 10.1021/acscentsci.3c00856 (PMC10540286; doi:10.1021/acscentsci.3c00856)
Supplement: Supplementary file 1 — oc3c00856_si_001.pdf [file oc3c00856_si_001.pdf]

Supporting Information

Wireless Electrochemical Reactor for Accelerated Exploratory Study of Electroorganic Synthesis

Jie Chen, and Yiming Mo*

Jie Chen - College of Chemical and Biological Engineering, Zhejiang University, Hangzhou, 310027, Zhejiang, China.

Yiming Mo - College of Chemical and Biological Engineering, Zhejiang University, Hangzhou, 310027, Zhejiang, China; ZJU-Hangzhou Global Scientific and Technological Innovation Center, Zhejiang University, Hangzhou, 311215, China.

Email: yimingmo@zju.edu.cn.

Table of contents

1. General information S3

2. Electronic circuit design and testing S3

3. Wi-eChem design S8

4. 3D printing procedure S11

5. Simulation study of mass transfer for Wi-eChem system S12

6. Luminol electrochemiluminescence visualization experiment S15

7. Electrooxidation of potassium ferrocyanide S16

8. Electrochemical decarboxylation S17

9. Electrochemical olefin-ketone coupling reaction S22

10. Automation platform (software) S26

11. Automation platform (hardware devices) S33

12. Nickel catalyzed oxygen atom transfer (OTA) reaction S42

13. References S44

1. General information

All the fluidic connections were achieved using standard 1/4-28 threaded fittings (https://www.idex-hs.com/) (https://www.runzefluidsystem.com/). Customized components were manufactured using 3D printing, CNC and laser cutting. All commercially available chemicals reagents and solvents were used as received from Aladdin and Sigma-Aldrich. All ^{1}H NMR were measured on a 400 MHz Bruker NMR instrument with the sample diluted by $CDCl_3$. Gas chromatography analysis was conducted on Agilent 8890 GC system with an FID detector. High performance liquid chromatography analysis was conducted on Agilent 1260 Infinity II HPLC system.

2. Electronic circuit design and testing

MATLAB Simulink simulation

Figure S1 shows the established wireless power transfer (WPT) model. DC Voltage Source module is used to simulate DC power. MOSFET module, Logical Operator module, Pulse Generator module are used to simulate the high-frequency inverter circuit. Mutual Inductance module is used to simulate the coupling coils. Capacitance module is used to simulate compensation network. Diode module and Capacitance module are used to simulate the rectifier circuit. Module parameters are set according to the parameters and related formulas of the actual circuit components (See Table S1 for detailed parameters).

The coil's inductance is 20 μH, the compensation capacitance is 150 nF, and the frequency of the circuit is 91.9 kHz according to the LC oscillation formula:

$$f = 1/\left(2\Pi\sqrt{LC}\right)$$

where f is the circuit frequency (Hz), L is the primary or secondary capacitance (F), and C is the transmitting or receiving coil's self-inductance (H).

The self-inductance of the transmitter coil and the receiver coil are both 20 μH. Since the coil shape is circular, the equivalent radius of the transmitter coil and the receiver coil are 5 mm and 1.5 mm, while their outer radius are 10 mm and 5 mm and their inter radius are 8 mm and 4.5 mm, respectively. The distance between the coils is 5 mm. According to the coil mutual inductance estimation formula,[1] the coupling coefficient is 0.065 and the mutual inductance is about 1.3 μH:

$$k = 1/\left[1 + 2^{2/3}\left(\frac{D}{\sqrt{r_1 r_2}}\right)^2\right]^{3/2}$$

$$L_m = k\sqrt{L_1 L_2}$$

where k is the coupling coefficient, D is the coil distance (mm), $r_1,\ r_2$ are the equivalent radius of transmitter coil and receiver coil (mm), $L_m$is the mutual inductance (H), and $L_1,\ L_2$ are the self-inductance of transmitter coil and receiver coil (H).

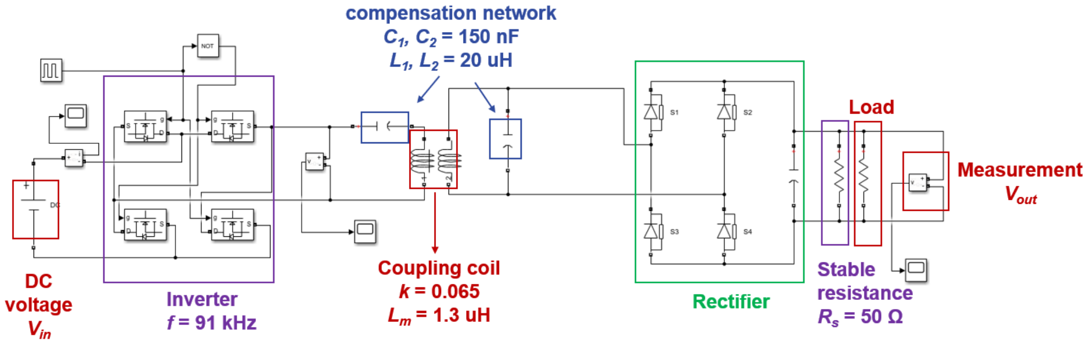

Figure S1. The WPT circuit model.

Table S1. Simulink modules and parameters

Module name	Parameter name	Parameter value
DC Voltage Source	Input voltage (V_{in})	0-3 V
MOSFET		
Logical Operator	Status	NOT
Pulse Generator	Frequency (f)	91.9 kHz
	Transmitter self-resistance (R1)	0.7 Ω
	Transmitter self-inductance (L1)	20 μH
Mutual Inductance	Receiver self-resistance (R2)	0.5 Ω
	Receiver self-inductance (L2)	20 μH
	Mutual inductance (Lm)	0.8 μH
	Primary capacitance (C1)	150 nF
Capacitance	Secondary capacitance (C2)	150 nF
	Filter capacitance (CL)	220 uF
Diode		
Stable resistor	Resistance (Rs)	50 Ω
Voltage measurement	Output voltage (V_{out})	

PCB design

Printed Circuit Board (PCB) are commercially available through Lichuang EDA (https://lceda.cn/) and Jialichuang (https://www.jlc.com/).

Figure S2 shows the PCB design of the wireless charging circuit. The transmitter part contains IRS2543 full-bridge drive circuit and IRF640B MOSFET inverter circuit. The receiver part contains SOT143 packaged BAS4002ARPPE6327HTSA1 chip as the rectifier bridge, C1206 packaged filter capacitor, R0805 packaged resistor as the voltage stabilizer, and C0805 packaged secondary compensation capacitor. All electronic components were purchased from LC@SC (https://www.szlcsc.com/).

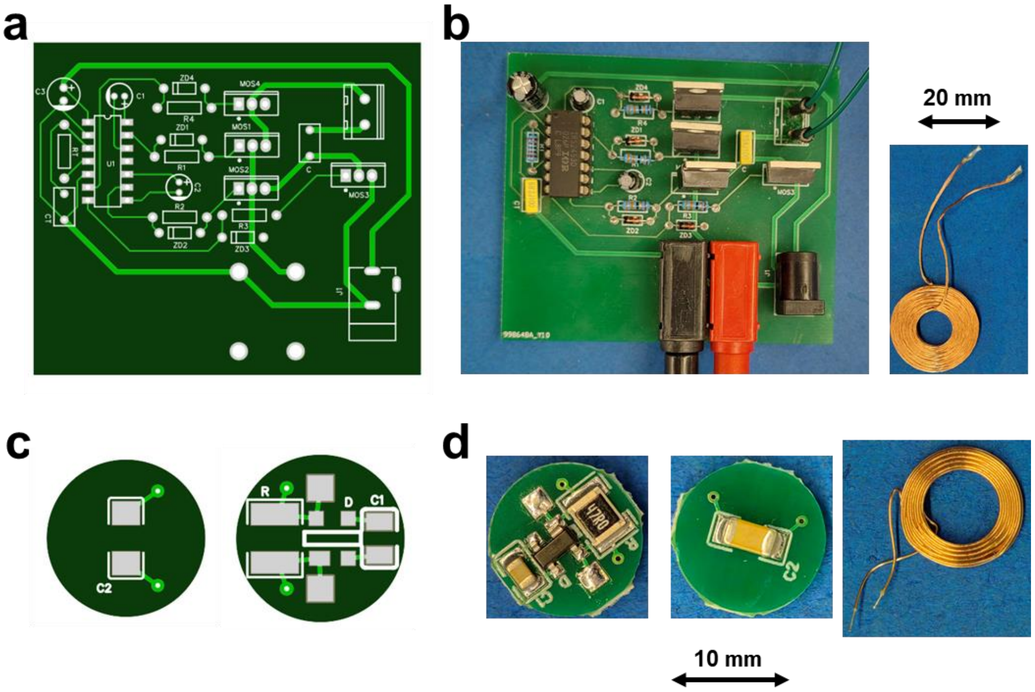

Figure S2. The PCB design of the wireless charging circuit. a) The transmitter circuit PCB design. b) The photo of transmitter circuit board. c) The receiver circuit PCB design. d) The photo of receiver circuit board.

Electronic circuit measurement

In order to avoid the influence of the rotation magnetic field used in magnetic stirring on the circuit, we used an ultra-high frequency design of 91 kHz and performed a rotation magnetic field test, and the results showed that the rotation magnetic field had no effect on wireless power transmission (Figure S3).

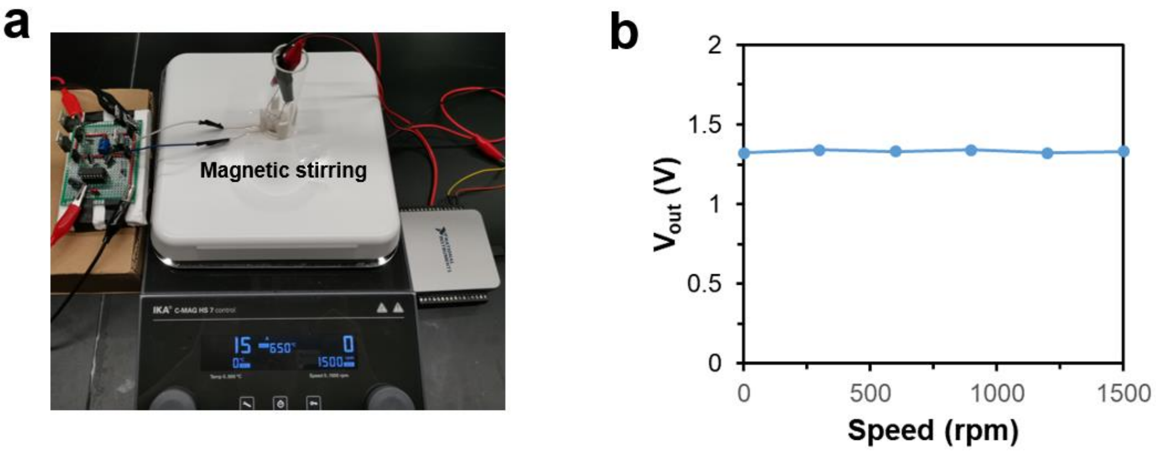

Figure S3. a) The setup for the rotation magnetic field testing. b) The data of output voltage at different rotation speed of the magnetic field for driving magnetic stirrer.

We also carried out output voltage measurement with different reaction electrolyte solutions to test output voltage stability and controllability. The test results showed that the voltage stabilization design in this circuit can make the output voltage variation range less than 0.3 V under the same input voltage. The curves under four different electrochemical reaction systems fall between 190 Ω and 2,000 Ω, which proved the ability of Wi-eChem for constant voltage operation.

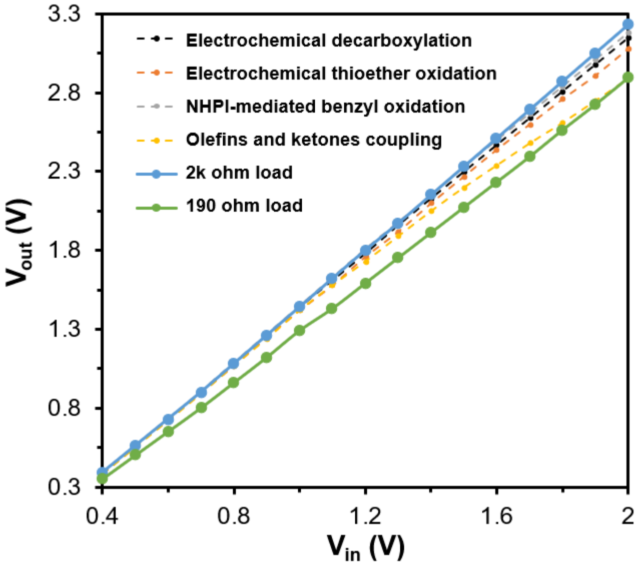

Figure S4. The calibration curve of V_{out} and V_{in} in different electrochemical reaction systems or resistor load.

3. Wi-eChem design

Wi-eChem contains two parts: a magnet stirrer (Wi-eChemStir) and a wireless charging base (Wi-eChemBase). The assembled CAD design and photo of the Wi-eChemBase is shown in Figure S5.

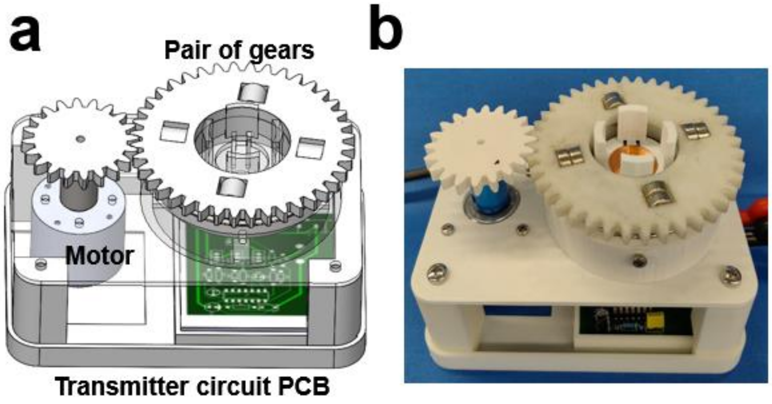

Figure S5. The design of the Wi-eChemBase. a) Exploded view. b) Photo of the Wi-eChemBase.

The detailed dimensions and photos of the main components in the Wi-eChemBase are shown as follows:

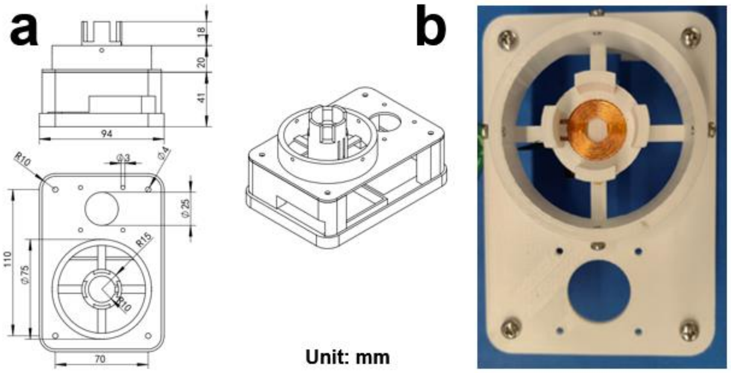

Figure S6. The support part of the Wi-eChemBase. a) CAD drawings with detailed dimensions. b) Photo of the support part.

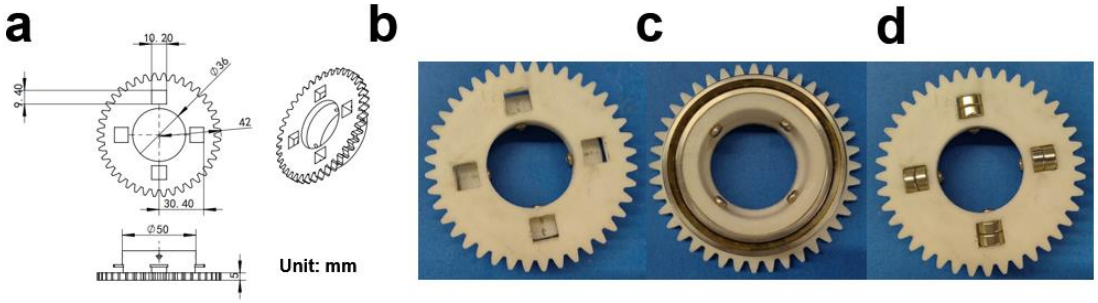

Figure S7. a) CAD drawings of the big gear with detailed dimensions. b) Photo of the big gear. c) Photo of the big gear with bearing (316L, 6810) installed. d) Photo of the big gear with four magnets (10×5 mm) installed.

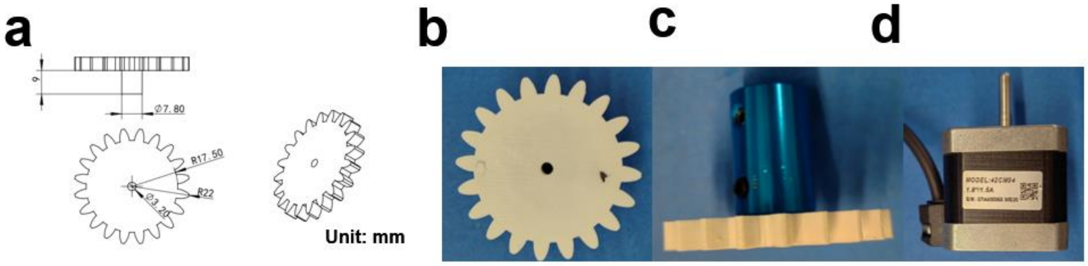

Figure S8. a) CAD drawings of the small gear with detailed dimensions. b) Photo of the small gear. c) Photo of the big gear with coupling (5 mm to 10 mm) installed. d) Photo of the motor (42CM04).

The assembled CAD design and photo of the Wi-eChemStir is shown in Figure S9. On the basis of the structure shown in Figure S9a, the assembled CAD design and photo of the parallel electrode setup for the reaction sensitive to the current density distribution is shown in Figure S9c-d. The electrical contact of the vertically mounted electrodes is achieved by two threaded holes in a PP cylinder with two M2 stainless steel screws inside, which can be installed directly on the two screws of the Wi-eChemStir. The reaction results for electrochemical decarboxylation using this parallel electrode setup are shown in Section 8.

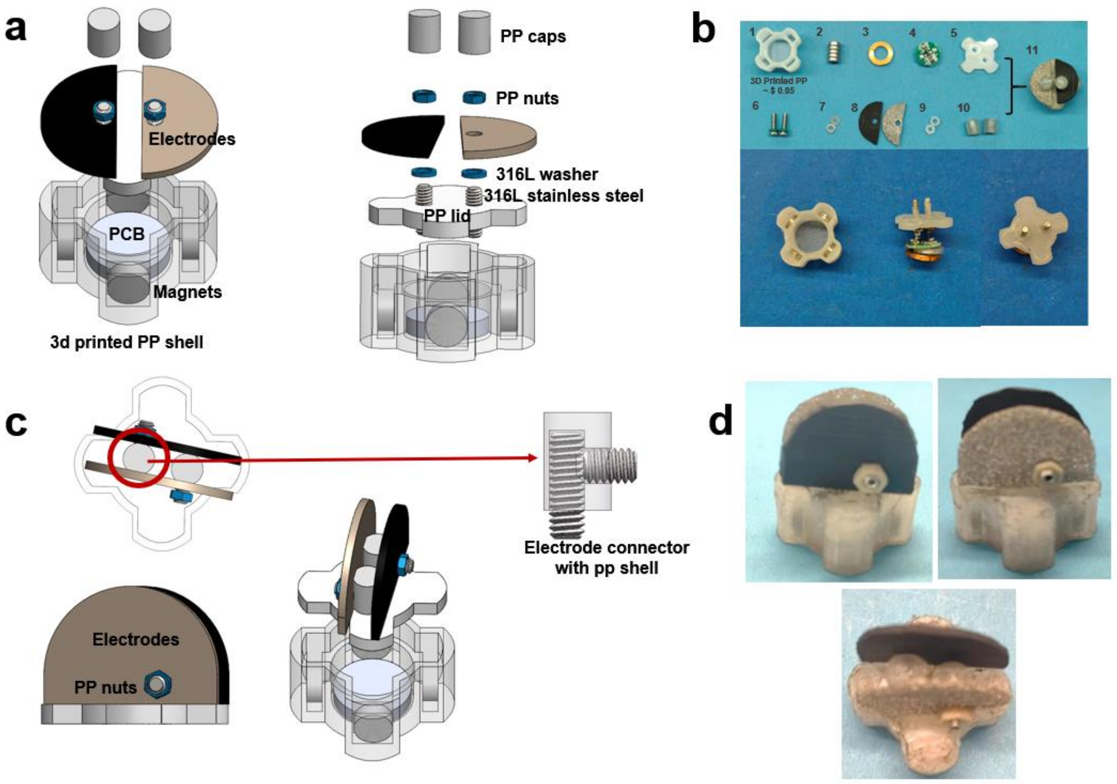

Figure S9. The design of the Wi-eChemStir. a) Exploded view. b) Photo of the components of the Wi-eChemStir and assembly method (1: 3D-printed PP shell, 2: magnets, 3: receiver coil, 4: receiver PCB, 5: PP lid, 6-7: 316L SS current collectors, 8: electrodes, 9: PP nuts, 10: PP caps, 11: Wi-eChemStir with electrodes installed). c) Exploded view and design details of the parallel electrode setup. d) Photo of the parallel electrode setup.

The details of the main components in the Wi-eChemStir are shown as follows:

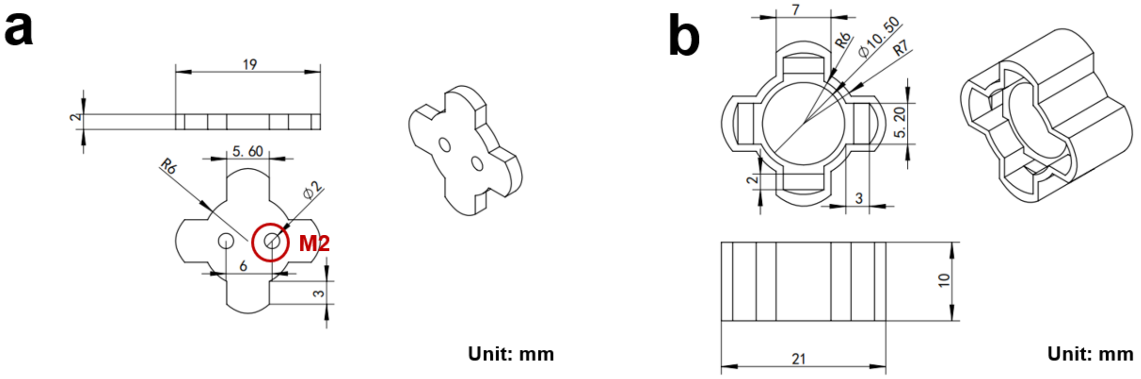

Figure S10. a) CAD drawings of the PP lid with detailed dimensions. b) CAD drawings of the 3D-printed PP shell with detailed dimensions.

The Wi-eChemStir is assembled as follows: (1) Install 4 magnets (5×2 mm) in the corresponding position of the PP shell (3D-printed); (2) Install 316L stainless steel (M2×8) wrapped with raw meal tape in the M2 threaded hole of the PP lid; (3) Connect the receiver coil, receiver PCB and 316L SS current collector using soldering; (4) Assemble the PP lid and PP shell; (5) Fuse the PP lid where it comes into contact with the PP shell with an electric soldering iron (The contact PP is melted and adhered together, Figure S11); (6) Install the 316L washer, electrodes, PP nuts and PP caps. PP cap is made as follows: M2 non-penetrating threaded holes are made in the center of a PP cylinder with a diameter of 4 mm.

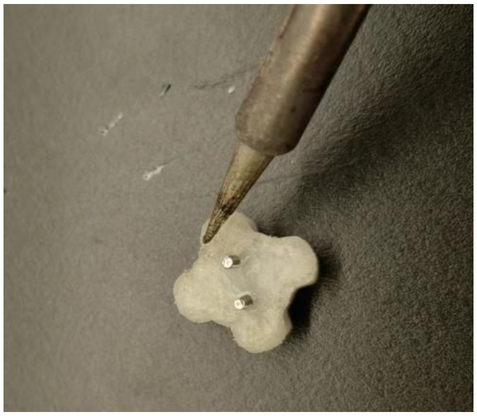

Figure S11. The PP shell and lid were melted and adhered together.

4. 3D printing procedure

First export the SOLIDWORKS model as STL format. Then use ideaMaker software to generate sliced models. Finally, use the RAISE3D E2 printer to print the model. The 3D printing slice parameters of PLA and PP materials are shown in Figure S12.

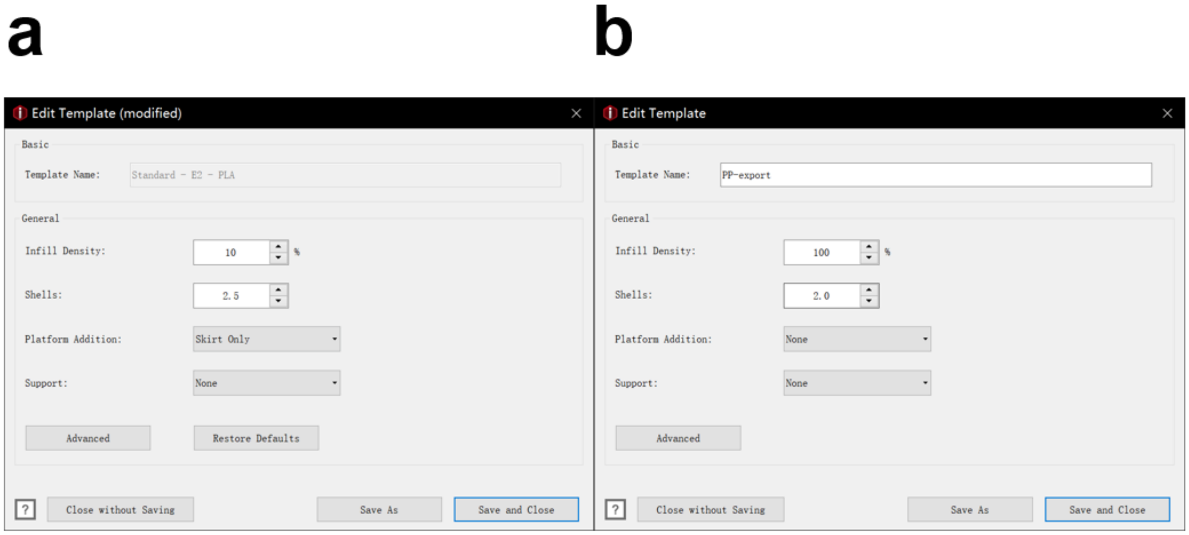

Figure S12. The 3D printing slice parameters of a) PLA and b) PP materials.

5. Simulation study of mass transfer for Wi-eChem system

The finite element analysis was used to study mass transfer behavior of Wi-eChem (Figure S13a, electrode rotation) and normal electrochemical reactors (Figure S13b, impeller rotation).

1. Geometry configuration

The geometry and dimensions were constructed according to Wi-eChem setup developed in this work. The mass transfer on the electrode surface is the focus of this analysis. Therefore, the red frame portions of Figure S13a and Figure S13b were used for geometry building.

As shown in Figure S13c, a cylinder with a height of 2 mm and a diameter of 25 mm is the stirrer electrode. The two semicircle boundaries on the bottom of the cylinder are the surfaces for anode and cathode.

2. Electrolyte domain configuration

A simple reaction, in which species A loses one electron at the anode surface to generate species B and species B gets one electron at the cathode surface to form species A, was used in the simulation.

Anode: A - e → B

Cathode: B + e → A

At the same time, in order to explore the influence of mass transfer effect, a high driving potential was applied, such that this reaction operates under mass transfer limited region. This is equivalent to the concentration of species A on anode surface is zero, and the concentration of species B on cathode surface is also zero.

2.1 Species diffusion and concentration

The diffusion coefficients of two species (A and B) were set in the domain with as 1e^{-9} m^2/s. The initial concentration of both species are set to 100 mol/m^3, and the average concentration during the entire simulation process are 100 mol/m^3.

2.2 Velocity field for Wi-eChem reactor

In Wi-eChem system (Figure S13a), the rotation of electrodes flings the solution outward from the center in a radial direction caused by the centrifugal force, in return, dragging the upper fluid towards its surface. Thus, the electrode surface is replenished by a flow normal to the surface.[2] The velocity in **r** direction and **z** direction was set according to Levich equation[2] since the Wi-eChem is similar to the rotating disk electrode.

$$U_z = -0.51\omega^{3/2}\nu^{-1/2}z^2$$

$$U_r = 0.51\omega^{3/2}\nu^{-1/2}rz$$

where ω is the angular velocity (1/s) and ν is the kinematic viscosity (m^2/s).

Considering that the influence of the electrode rotation on the reaction liquid will gradually decrease with the distance from the electrode, the velocity in **φ** direction decreases linearly from the electrode according to the Newtonian law of viscosity.

$$U_\varphi = \omega r\left(\frac{z}{2[mm]}\right)$$

2.2 Velocity field for the normal electrochemical reactor

For normal electrochemical reactors (Figure S13b), the magnetic stirring generates a rotating velocity field. The mass transfer is improved by the thickness reduction of the diffusion boundary layer on the electrode surface. Therefore, we assume that U_r and U_z in normal electrochemical reactor are 0. The U_φ remains the same as the Wi-eChem setup.

3. Boundary conditions

The boundary conditions for Wi-eChem and normal reactor are shown in Table 2.

Table 2. Boundary conditions

**Boundary**	**Mass transfer equation**	**Description**
1	$c_A = 0$	A fast-irreversible reaction (A→B) with the concentration of A is 0.
2	$c_B = 0$	A fast-irreversible reaction (B→A) with the concentration of B is 0.
3	$-n \cdot J_i = 0$	No flux.
4	$-n \cdot J_i = 0$	No flux.
5	$-n \cdot J_i = 0$	No flux.

Based on the above conditions, the flux of A at the anode surface can be calculated. The electrochemical current is proportional to the flux at the electrode surface[2]:

$$i_A = \mathrm{nFD} \int (\frac{\partial C_A}{\partial \mathrm{z}})_{z=0}$$

The relationship curves between current i_A and angular velocity ω were plotted (Figure S13d). The results show that the current of Wi-eChem is proportional to 1/2 power of the angular velocity. In the normal electrochemical reactor, the current is proportional to 1/3 power of the angular velocity.

$$i_{Wi-echem} \sim \omega^{1/2}$$

$$i_{normal} \sim \omega^{1/3}$$

The mass transfer of Wi-eChem setup shows a stronger dependence on the stirring speed than that of the normal electrochemical reactor. Thus, Wi-eChem setup can benefit more from increasing the stirring speed.

To be noted, this simulation study only considered the extremely simiplied scenario. In real conventional electrochemical reactors, the actual mass transfer behavior depends on the shape of the impeller and the geometric locations of electrodes relative to the impeller. The actual dependence of mass transfer limiting current on the rotating speed ω for normal electrochemical reactor can be between $\omega^{1/3}$~$\omega^{1/2}$.

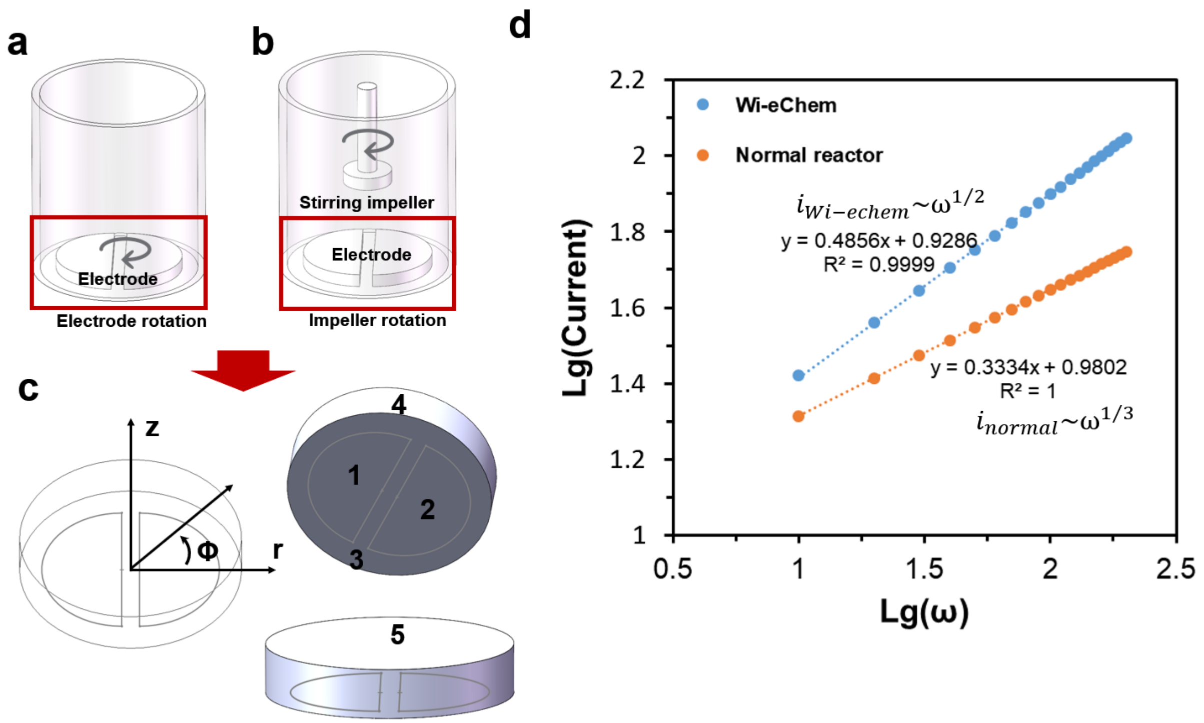

Figure S13. a) Wi-eChem: electrode rpotation. b) Normal electrochemical reactor: impeller rotation. c) The geometry built for finite element analysis. d) The current of Wi-eChem is proportional to 1/2 power of the angular velocity. e) The relationship curves between current i_A and angular velocity ω.

6. Luminol electrochemiluminescence visualization experiment

For the luminol electrochemiluminescence reaction, the carbon paper (18 mm diameter semicircle) was used as anode and cathode. The glass vial was charged with sodium carbonate (0.1 M), sodium bicarbonate (0.1 M), luminol sodium salt (0.05 M), hydrogen peroxide (1.5 M), water (5 ml) and the Wi-eChemStir equipped with electrodes. The electrolysis voltage was set as 3.0 V.[3] Then, the vial was placed on the Wi-eChemBase for the following reaction. During the reaction, the anode surface emitted blue light (Figure S14).

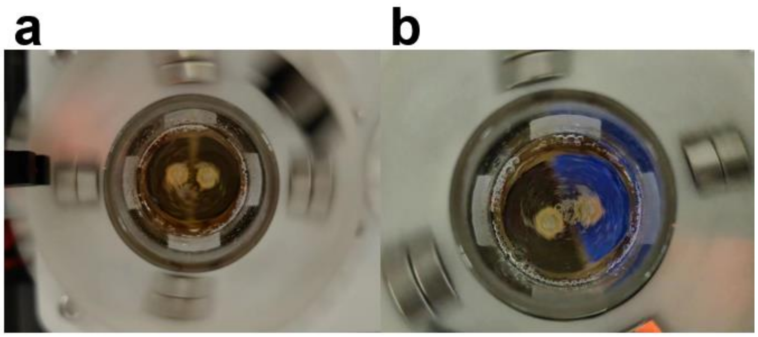

Figure S14. a) Power off. b) Power on.

7. Electrooxidation of potassium ferrocyanide

The construction diagram of the absorbance measurement device is shown in Figure S15b-c. The liquid circulation was realized using the peristaltic pump and the flow pipeline, and the inline detection of the solution absorbance at the pump outlet was realized through the UV-Vis spectrometer (HR2000+, Ocean Optics).

Potassium ferricyanide (0.025 M) aqueous solution and potassium ferrocyanide (0.025 M) aqueous solution were prepared. The two solutions were injected into the flow pipeline to obtain the absorbance curves (Figure S15a).

According to Figure S15a, potassium ferricyanide has an absorbance peak at 420 nm, but potassium ferrocyanide has a neglible absorbance at 420 nm, so the inline detection wavelength of absorbance was set as 420 nm.

For the electrochemical oxidation of potassium ferricyanide, the metal Ni foam (18 mm diameter semicircle) was used as anode and cathode. The glass vial was charged with potassium ferrocyanide (0.1 M), water (10 ml), and the Wi-eChemStir equipped with electrodes. Then the vial was placed on the Wi-eChemBase for the following reaction.

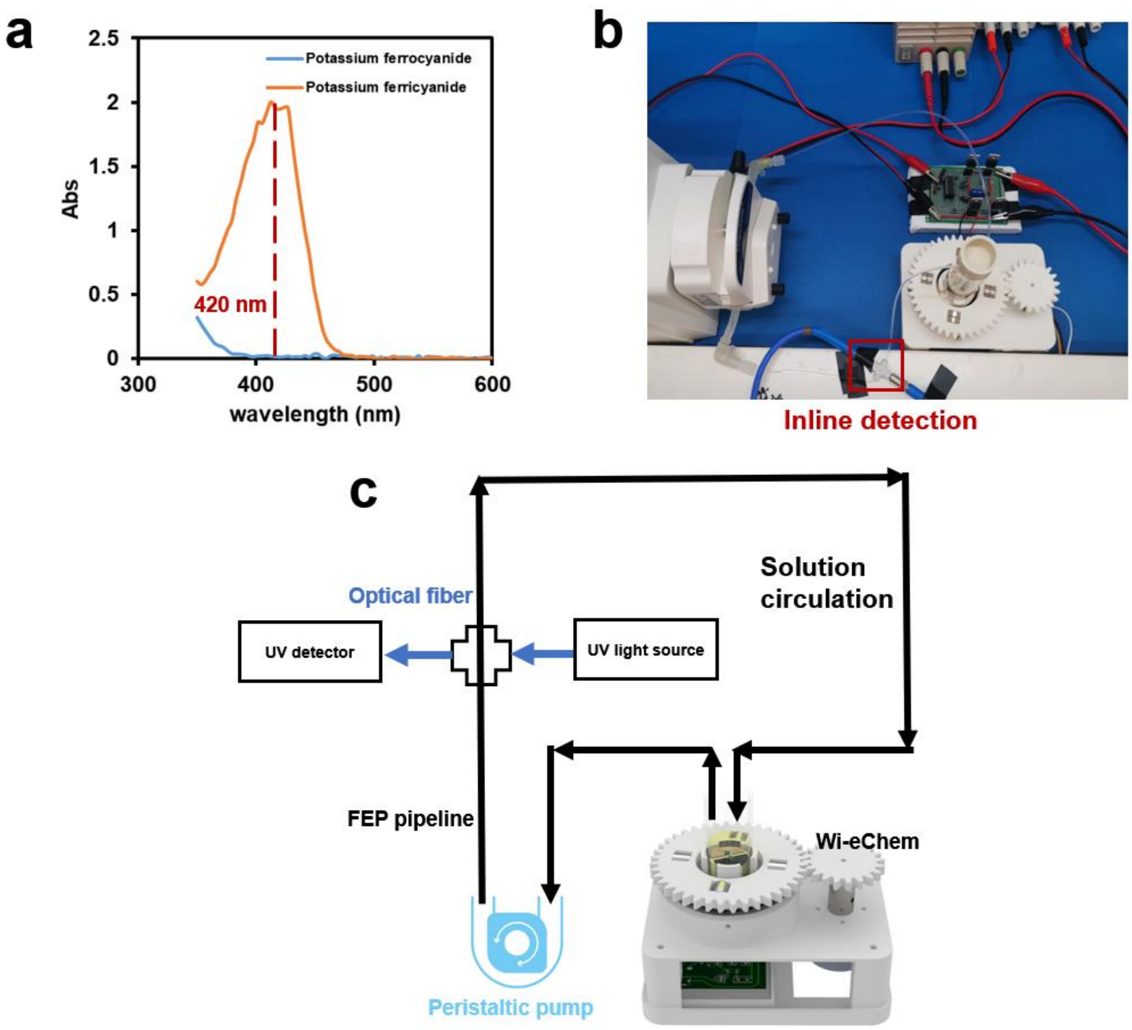

Figure S15. a) The absorbance spectrum of potassium ferrocyanide and potassium ferricyanide. b) The construction diagram of the absorbance measurement device. c) Setup diagram for inline measurement of solution absorbance.

8. Electrochemical decarboxylation

Electrochemical decarboxylation with NaOH as base

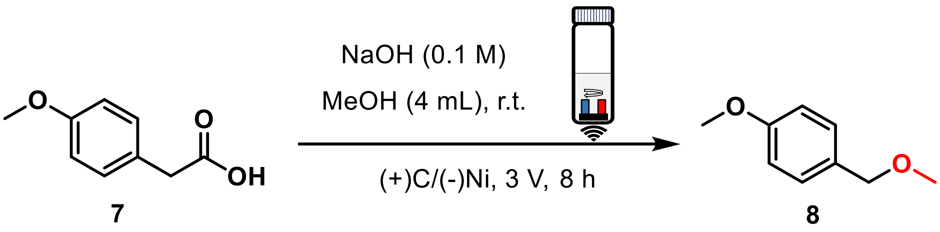

For the electrochemical decarboxylation with NaOH as base, the graphite plate (18 mm diameter semicircle) was used as the anode, the metal Ni foam (18 mm diameter semicircle) was used as the cathode. The glass vial was charged with 4-methoxyphenylacetic acid (**7**, 0.1 M), NaOH (0.1 M), methanol (4 mL) and the Wi-eChemStir equipped with electrodes. The electrolysis voltage was set as 3.0 V, the rotation speed was set as 150rpm. Then the vial with lid closed was placed on the Wi-eChemBase for the following reaction. After 8 h electrolysis, mesitylene (0.07 mmol, 10 μL) was added as internal standard. Then the reaction solution was removed from the vial, the vial and the Wi-eChemStir were cleaned three times with MeOH, the cleaning solution and reaction solution were combined. Solvent methanol was removed with rotovap. EtOAc and water were added for extraction, and then the upper organic phase was dried with anhydrous $MgSO_4$. The reaction yield was determined by GC. The GC calibration curve for determining the yield of target product **8** is shown in the Figure S16.

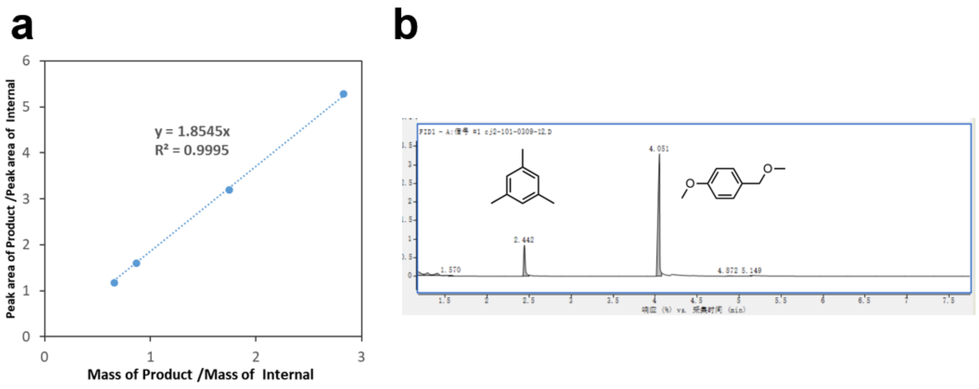

Figure S16. a) GC calibration curve for determination of yield of **8**. b) GC chromatograph of **8** and internal standard.

By using the parallel electrode setup demonstrated in Section 3 and the same procedure described above, five repeated experiments conducted with the same Wi-eChemStir (parallel electrode setup) gave stable yields. The reaction performance was the same as the non-parallel electrode setup.

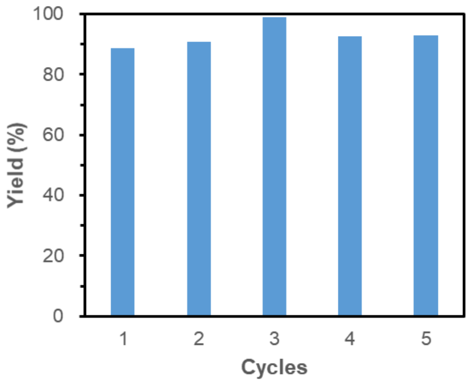

Figure S17. Yield of the five repeated experiments.

Reaction under different rotation speeds

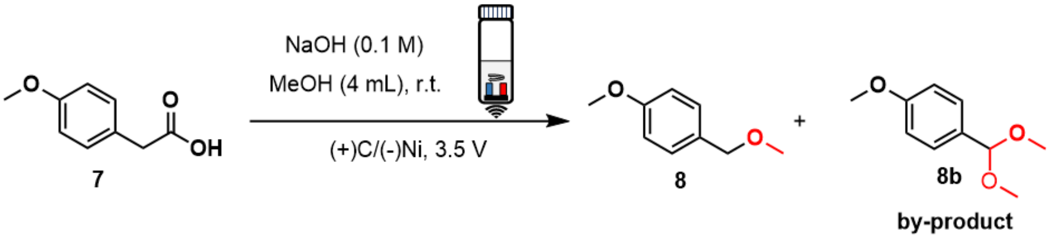

For the reaction under different rotation speed, the graphite plate (18 mm diameter semicircle) was used as the anode, and the metal Ni foam (18 mm diameter semicircle) was used as the cathode. The glass vial was charged with 4-methoxyphenylacetic acid (**7**, 0.1 M), NaOH (0.1 M), dodecane (20 μl, internal standard), methanol (4.5 mL) and the Wi-eChemStir equipped with electrodes. The electrolysis voltage was set as 3.5 V. The rotation speed was set as 10 rpm, 50rpm, 150rpm, and 200 rpm. 50 μl samples were acquired at 0.5 h, 1 h, 1.5 h, 2 h, 2.5 h, 3 h, 4 h, 5 h, and 6 h, and the yield of product **8** and main by-product **8b** were determined using GC. The result is shown in Figure S18.

From Figure S18, at lower rotation speed (10 rpm and 50 rpm), the reaction tends to produce more by-product **8b**, while at higher rotation speed (150 rpm and 200 rpm), the reaction gave higher selectivity towards the target product **8**. The production of by-product at lower rotation speed mainly due to the fact that the mass transfer rate cannot match the reaction rate on the electrode surface. Since the target product **8** on the anode surface cannot leave quickly through the rotation of electrodes, it is easy to be overoxidized to **8b** under higher voltage. While at higher rotation speed, reactant **7** can supplied to the electrode surface in a timely manner.

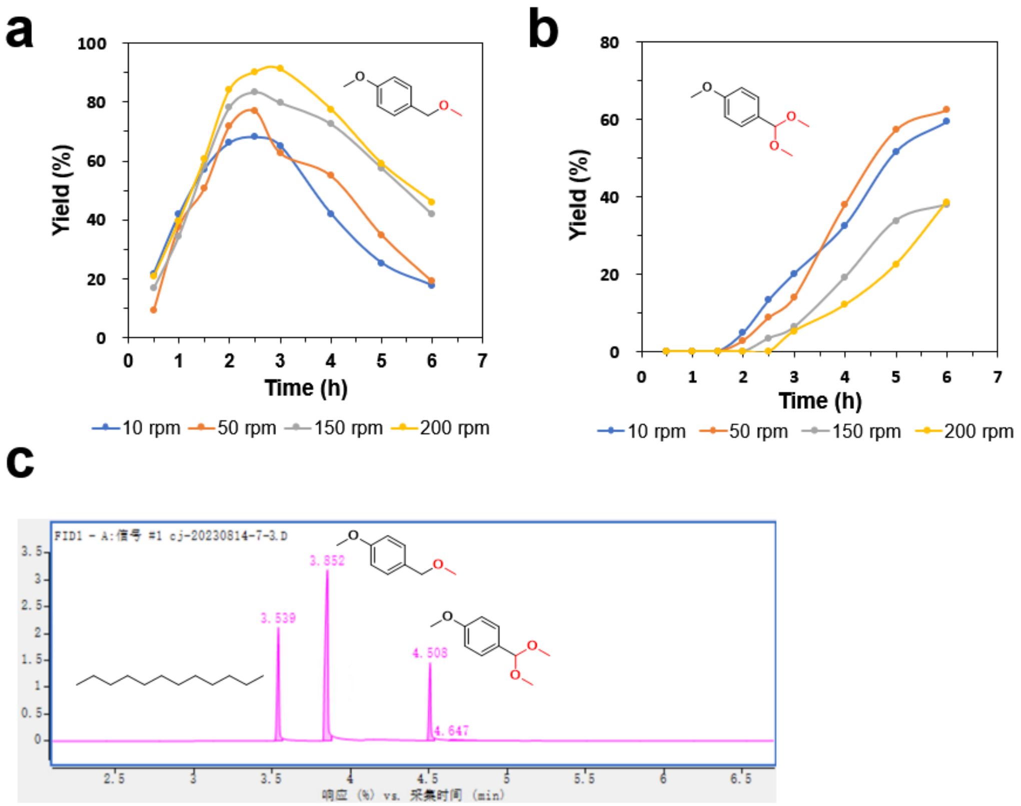

Figure S18. a) Yield of product **8**. b) Yield of by-product **8b**. c) GC chromatograph of **8**, **8b** and internal standard.

Electrochemical decarboxylation using the silica-supported base

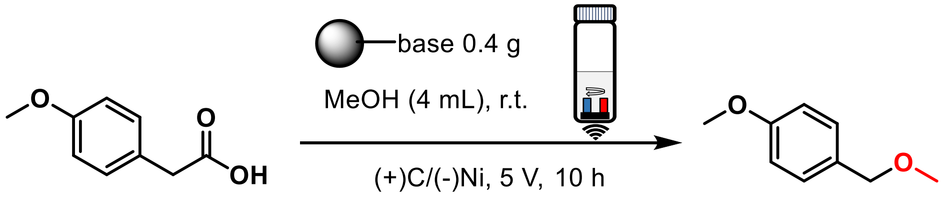

7 **8**

For the electrochemical decarboxylation, the graphite plate (18 mm diameter semicircle) was used as the anode, the metal Ni sheet (18 mm diameter semicircle) was used as the cathode. The glass vial was charged with 4-methoxyphenylacetic acid (**7**, 0.1 M), silica-supported base (0.4 g, Figure S19), methanol (4 mL) and the Wi-eChemStir equipped with electrodes. The electrolysis voltage was set as 5.0 V[4] , the rotation speed was set as150 rpm. Then the vial with lid closed was placed on the Wi-eChemBase for the following reaction. After 10 h electrolysis, mesitylene (0.07 mmol, 10 μL) was added as internal standard. A sample was taken and the reaction yield was

determined by GC using the same procedure described above. The silica-supported base was reused after filtration, sodium bicarbonate solution cleaning, ethanol cleaning to pH = 7, and drying in a vacuum oven at 60 °C.

The synthesis of silica-supported base

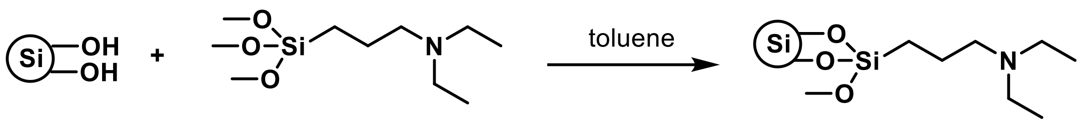

5.0 g of SPE silica gel (the particle and pore size of the SPE padding silica were 0.05 mm diameter and 10 nm, respectively) was suspended in 50 mL of dry toluene, and 5 mmol (N, N-Diethyl-3-aminopropyl) trimethoxysilane was added to this suspension. The mixture was refluxed under dry nitrogen atmosphere for 24 h at 110 °C. The modified silica gel was filtered off, washed twice with toluene, and dried under vacuum at room temperature before use.[5]

Organic elemental analysis of self-made silica-supported base

Measuring the nitrogen content of silica-supported base through organic elemental analysis (Thermo Scientific FlashSmart) can determine the molar amount of base loaded on silica gel per unit mass. The test results showed that during the cycle process, the molar amount of base loaded on silica gel gradually decreases. Since the current is related to the effective base equivalence during the electrochemical reaction, the conversion of the reaction decreases as the amount of base on silica drops (constant potential electrolysis with certain electrolysis time).

Table S3. Result of organic element analysis

**Cycle time**	**Nitrogen content**	**Molar amount of base**
0 (new)	6.296%	4.5 mmol/g
1	2.947%	2.1 mmol/g
10	1.408%	1.0 mmol/g

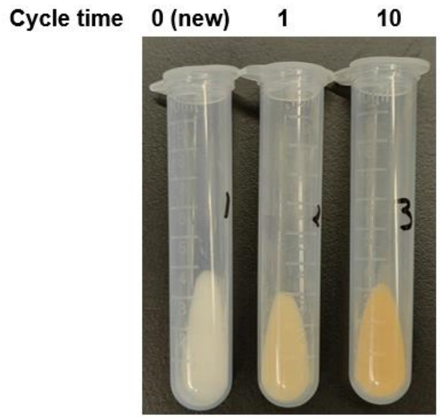

Figure S19. Photos of silica-supported base.

9. Electrochemical olefin-ketone coupling reaction

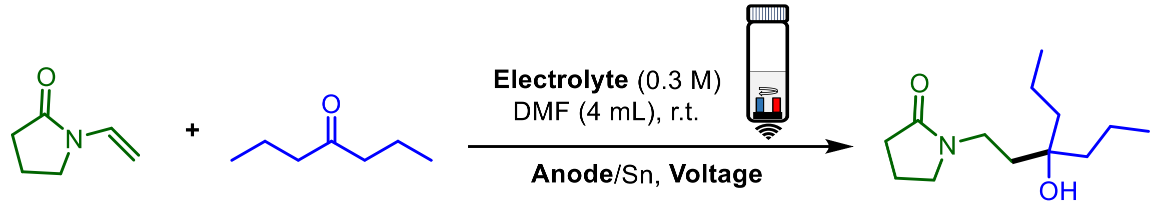

9 10 11

For the olefin-ketone coupling reaction, the sacrificial metal sheet (Zn, Al and Mg, 18 mm diameter semicircle) was used as the anode, the Sn sheet (18 mm diameter semicircle) was used as the cathode. The glass vial was charged with N-vinyl-2-pyrrolidone (**9**, 0.35 M), 4-heptanone (**10**, 0.7 M), electrolyte (Bu_4NBr, Bu_4NCl, Bu_4NClO_4, or Bu_4NBF_4, 0.3 M), DMF (4 mL), and the Wi-eChemStir equipped with electrodes.[6] Then the vial with lid closed was placed on the Wi-eChemBase for the following screening. After 16 h electrolysis, mesitylene (0.28 mmol, 40 μL) was added as the internal standard. Then, the reaction solution was removed from the vial, the vial and the Wi-eChemStir were cleaned three times with DMF, the cleaning solution and reaction solution were combined. A disposable dropper can be used as a column/filtration utensil and a piece of cotton can be used as a plug. A ~3 cm of silica gel was added, then ~0.5 mL reaction mixture was added neat onto the dry silica plug. After sample properly loaded on the silica, ~8 mL of EtOAc was added to perform a quick silica plug filtration.[6] After filtration, the filtered sample was washed by water, the upper organic phase was dried with anhydrous $MgSO_4$. The reaction

yield was determined by GC. The GC calibration curve for determination of yield of target product **11** is shown in Figure S20.

Purification via flash column chromatography (hexanes/EtOAc, 3:1 to pure EtOAc) afforded the targeted product **11** (Figure S21).

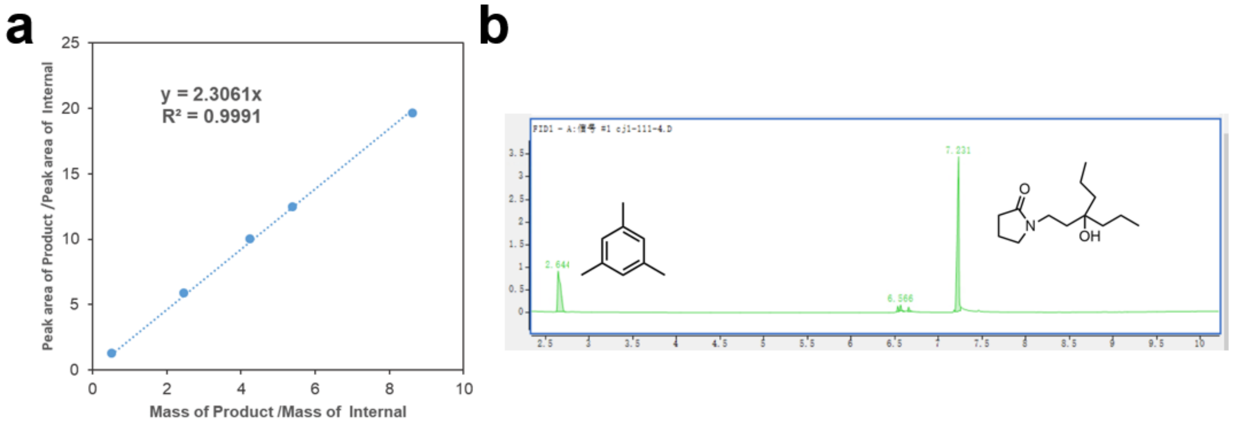

Figure S20. a) GC calibration curve for determination of yield of **11**. b) Gas chromatogram of **11** and internal standard.

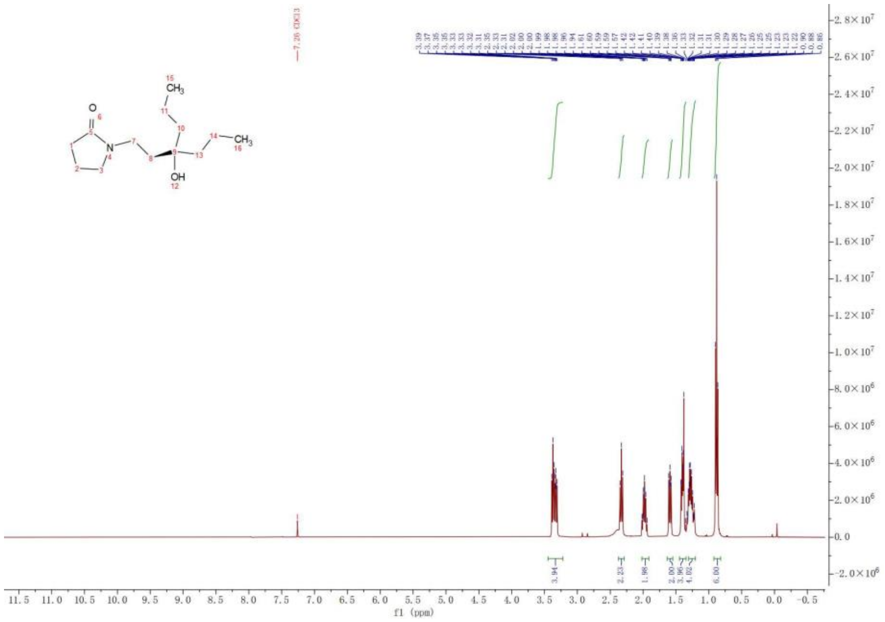

Figure S21. ^{1}H NMR of target product **11**.

Table S4. The screening results of the olefin-ketone coupling reaction (the reported yield in the main text is the average of three runs per reaction condition).

Reaction condition	Yield (%)
Zn/Bu_4NBr/2.0 V	67.5/65.5/76.0
Zn/Bu_4NCl/2.0 V	59.4/60.6/49.0
Zn/ Bu_4NClO_4/2.0 V	63.5/62.9/62.4
Zn/ Bu_4NBF_4/2.0 V	57.6/51.2/53.1
Zn/Bu_4NBr/2.5 V	74.4/71.6/65.9
Zn/Bu_4NCl/2.5 V	54.4/52.7/55.3
Zn/ Bu_4NClO_4/2.5 V	74.3/67.3/62.3
Zn/ Bu_4NBF_4/2.5 V	56.1/60.8/60.7
Mg/Bu_4NBr/2.0 V	50.4/44.1/46.2
Mg/Bu_4NCl/2.0 V	53.4/55.1/55.1
Mg/ Bu_4NClO_4/2.0 V	50.6/38.3/45.4
Mg/ Bu_4NBF_4/2.0 V	63.9/51.5/52.2
Mg/Bu_4NBr/2.5 V	40.5/44.2/41.1
Mg/Bu_4NCl/2.5 V	43.3/44.8/49.4
Mg/ Bu_4NClO_4/2.5 V	28.8/32.9/34.0
Mg/ Bu_4NBF_4/2.5 V	43.1/37.2/36.7
Al/Bu_4NBr/2.5 V	61.3/53.4/49.3
Al/Bu_4NCl/2.5 V	34.1/29.0/34.8
Al/ Bu_4NClO_4/2.5 V	0.5/0.2/0.1
Al/ Bu_4NBF_4/2.5 V	60.9/63.5/61.7
Al/Bu_4NBr/3.0 V	39.6/40.6/40.3
Al/Bu_4NCl/3.0 V	45.7/39.7/35.6
Al/ Bu_4NClO_4/3.0 V	5.6/0.1/0.1
Al/ Bu_4NBF_4/3.0 V	49.3/48.2/49.4

Among them, the combination of aluminum and Bu_4NClO_4 could hardly produce any target product. The main reason was that the precipitation generated by Bu_4NClO_4 and aluminum ion passivated the surface of the aluminum electrode, resulting in a decrease in conductivity. The precipitation is shown in Figure S22. The results of scanning electron microscopy also showed

that the passivated aluminum electrode caused image distortion, deformation, shaking and other phenomena due to poor conductivity (Figure S23).

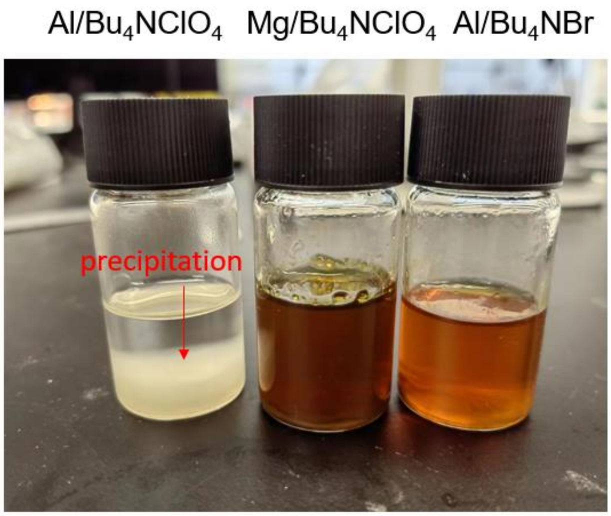

Figure S22. The reaction solutions after olefin-ketone coupling reaction.

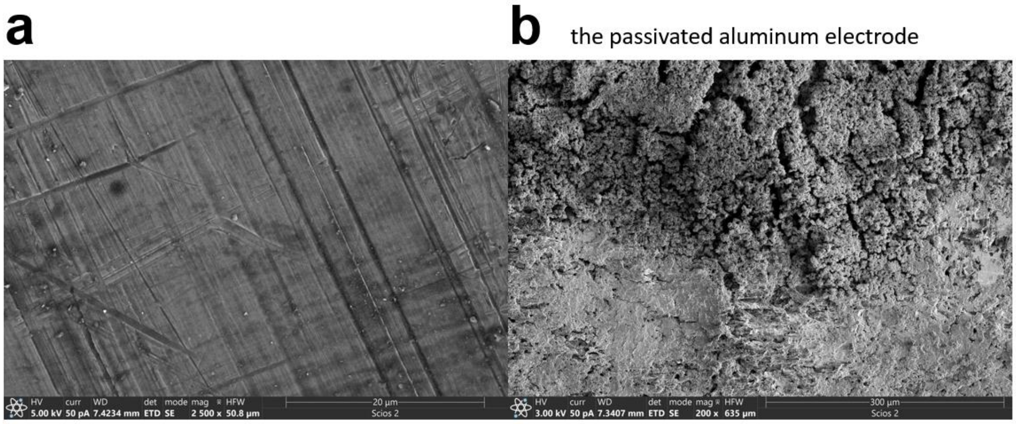

Figure S23. The results of scanning electron microscopy. a) Surface of a new aluminum sheet. b) Surface of aluminum sheet after the reaction using Al/Bu_4NClO_4 as the reaction condition.

10. Automation platform (software)

The software (https://github.com/handward/Wi-eChem-automation) is written in Python (execution back-end) and LabVIEW (graphical front-end, Figure S24). The Python part packs the operation steps and the parameters of each step into the form of JSON, and allows users to change procedure parameters (e.g., pipetting volume, reaction time, and electrolysis voltage) by submitting an Excel file. The LabVIEW part is responsible for reading the JSON commands generated by Python, providing user interaction interface, and communicating with the devices.

The JSON example is as follows:

```
{
    "number of process": 2,
    "experiment_No": 1,
    "process1": {
        "1": {
            "device": "public_device_occupy",
            "operation": {
                "ID": 1,
                "command": "robot, pump, relay",
            }
        },
        "2": {
            "device": "robot",
            "operation": {
            "ID": 1,
            "command": "x = 45.0, y = 0.0, z = -10.0, r = 0.0, speed = 100, rough = 0.0"
            }
        }
        "number of steps": 2
    },
    "process2": {
    "1": {
        "device": "power_supply1",
        "operation": {
            "ID": 1,
            "command": "2.11"
        }
    },
        "number of steps": 1
    }
}
```

JSON command contains four layers of information structure: (1) Parameters at the process level; (2) Parameters at the step level; (3) Parameters at the device/operation level; (4) Specific parameters transmitted to the device. A complete JSON command includes four processes (preparation, reaction, workup and cleaning).

Devices and their corresponding operations are shown in Table S5.

Table S5. Devices and their corresponding operations

Device	Operation	Description
Robotic arm (robot)	{"ID": 1, "command": "x = 45.0, y = 0.0, z = -147.13, r = 0.0, speed = 100, rough = 0.0"}	The robotic arm moves to the target point (45.0, 0.0, -147.13) at a speed of 100 (robotic arm custom speed), and the gripper's angle is 0.0°.
Syringe pump (pump)	{"ID": 1, "command": "channel = O, volume = 4.0, rate = 10.0, wait_time = 3.0"}	The syringe pump draws 4.0 mL of liquid at a speed of 10.0 mL/min, and then waits for 3.0 s.
Power supply (power_supply)	{"ID": 1, "command": "2.11"}	The set voltage of No. 1 power supply is 2.11 V.
Wi-eChem (motor)	{"ID": 1, "command": "block1"}	Wi-eChem #1 operates in block1 mode.
Wi-eChem (wait)	{"ID": 1, "command": 1}	Wi-eChem #1 electrochemical time is set to 1 h.
Mechanical Gripper (robot_efg)	{"ID": 1, "command": "distance = 14"}	The distance between grippers of the mechanical grippers is set to 14 mm.

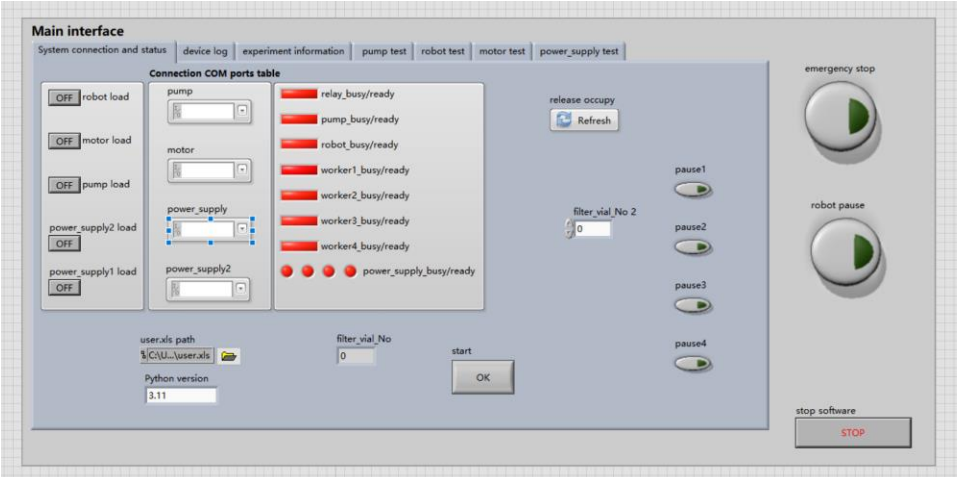

Figure S24. LabVIEW Graphical Interface.

	A	B	C	D	E	F	G	H	I	J	K	L	M	N	O
1	Experimen	unit(ml)												1 represents finished	
2		A	B	C	D	E	F	G	H	I	J	Voltage(V)	time(h)		
3	1	3				1						2.11	2	0	0
4	2		3			1						2.08	4	0	0
5	3			3		1						1.91	4	0	0
6	4				3	1						1.66	4	0	0
7															

Figure S25. The Excel file of experimental tasks and parameters.

Excel file defines experimental tasks and parameters (Figure S25), each row represents an experimental task, the N column uses 1/0 to indicate whether the task is completed, and the O column indicates the corresponding LC filter vial number after the experimental task is completed.

The software follows a "scheduler-worker" control scheme which includes 1 central scheduler and 4 workers. In this control scheme, the user submits the Excel file to the central scheduler, and then the scheduler allocates tasks to idle workers according to the order in Excel (start with the first unfinished experimental task). As shown in Figure S26 and Figure S27, in a task allocation loop, the central scheduler will only allocate one task to an idle worker in each cycle, and the assignment priorities of worker #1 to #4 are from high to low (Figure S27), that is, when worker #1’s status is busy, worker #2 is assigned to the task, then restart the task allocation loop or when worker #1’s and worker #2’s status are busy, worker #3 is assigned to the task, then restart the task allocation loop.

When a worker is assigned a task, the worker's state changes from idle to busy, and then restarts the task allocation loop, at the same time, the worker executes a preparation-reaction-workup-cleaning experiment, and then the status changes from busy to idle.

In addition, in this control scheme, before entering the task allocation loop, it is necessary to judge whether the experimental task is allocated. If the allocation is completed, wait for the user to submit new experimental tasks, and the task allocation loop will not run during the waiting period.

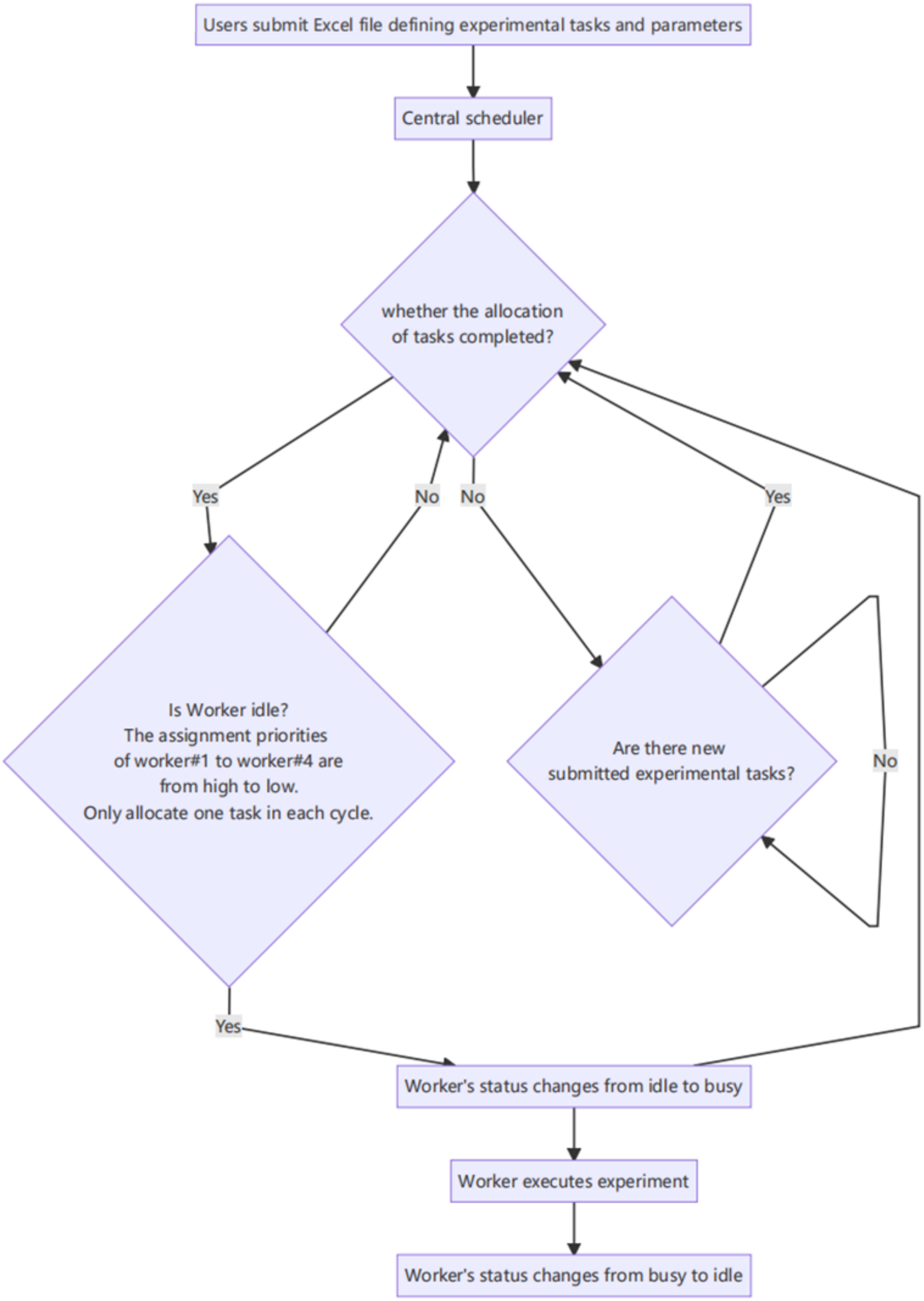

Figure S26. The "scheduler-worker" control scheme: simplified program diagram.

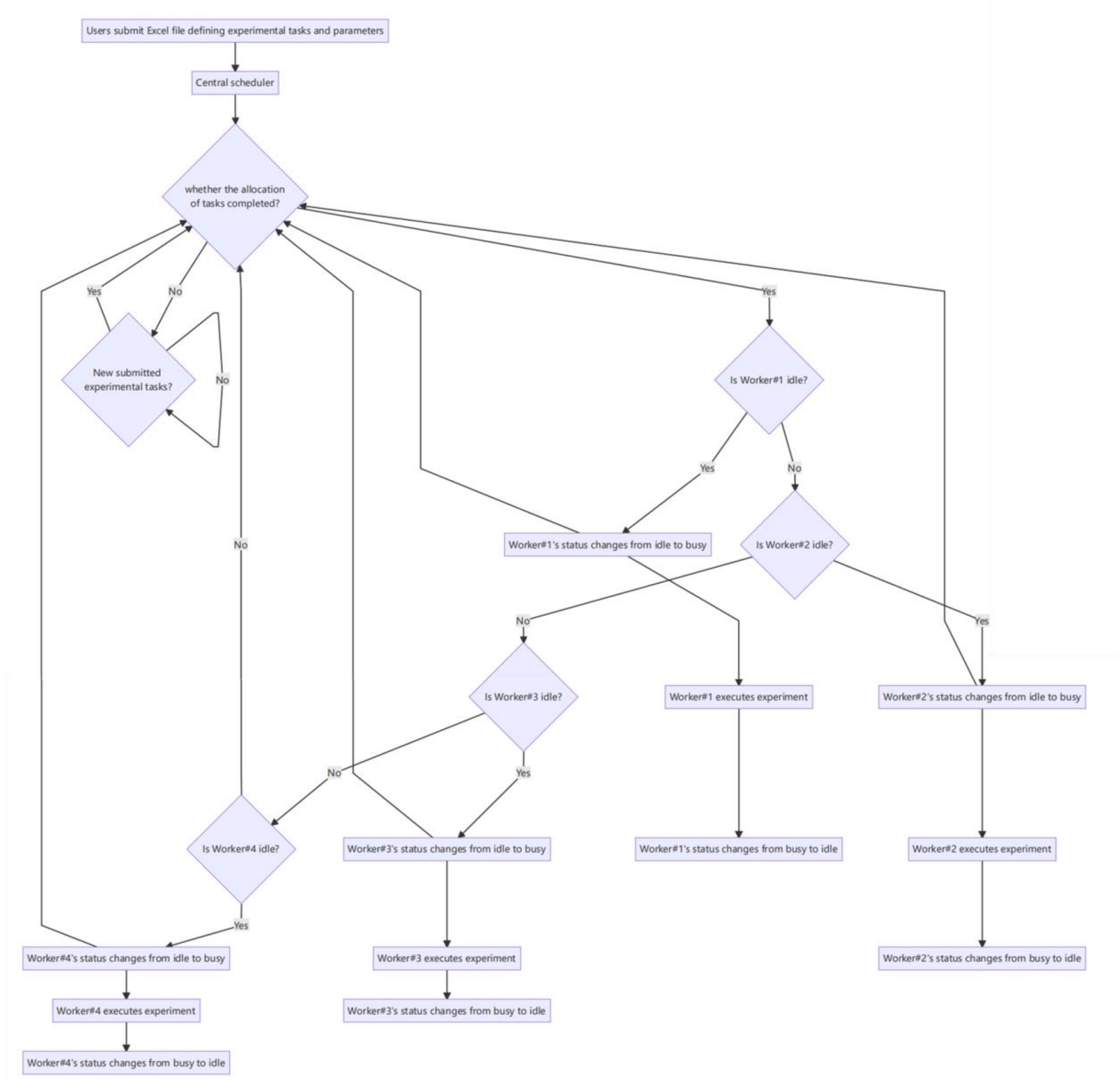

Figure S27. The "scheduler-worker" control scheme: complete program diagram which shows the assignment priorities.

When the worker executes the preparation-reaction-workup-cleaning experiment, since all 4 workers will be assigned to experimental tasks, in order to schedule 4 workers to achieve high utilization of the device, a first-come, first served (FIFS) queue is designed.

In this FIFS queue, the worker reads and unpacks JSON command generated by the Python back-end, and obtains the parameters of each process (preparation, reaction, workup, and cleaning). Before scheduling the required hardware modules (devices) to complete the subtask, the worker needs to enter the FIFS queue. If there are no other workers waiting in the FIFS queue, the worker will be released and enter the step of judging whether the required devices are idle, otherwise continue to wait. Then, when the required devices are all idle, the worker occupies these devices and completes the process, and finally releases these occupied devices.

When all processes are completed, that is, after cleaning is completed, work's status changes from busy to idle, otherwise continue to enter the FIFS queue.

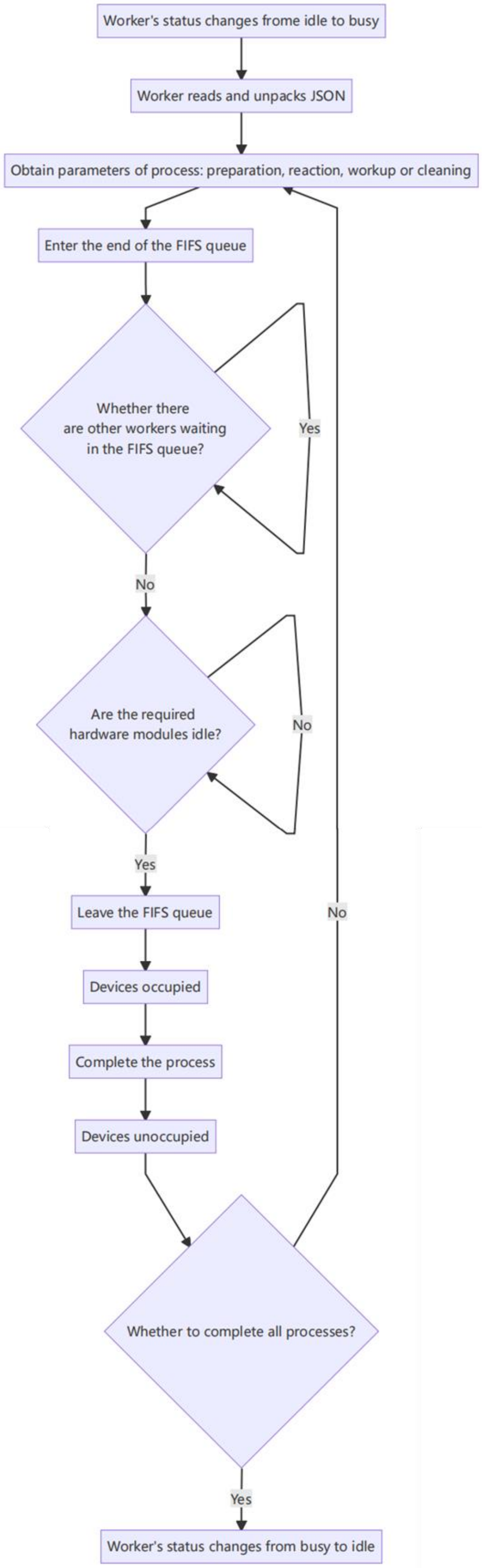

Figure S28. FIFS queue: complete program diagram.

11. Automation platform (hardware devices)

The hardware devices of automated platform are shown in Table S6, the photo of automated platform is shown in Figure S29, and the operation videos of automation platform are shown in Video S2:

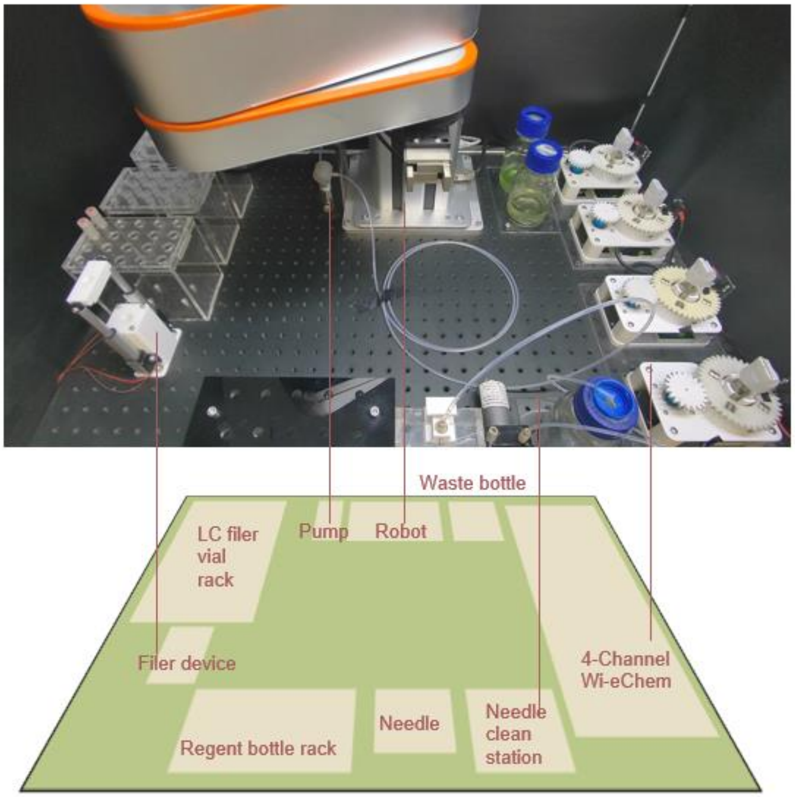

Figure S29. The photo of automated platform.

Table S6. The hardware devices of automated platform

Device No.	Device description	Model
01	Optical table for mounting and positioning various devices	
02	SCARA (robot) with an electric gripper installed	HITBOT Z-Arm 2442 & Z-EFG-20.
03	Syringe pump	LONGER MSP1-CX
04	Wi-eChemStir & Wi-eChemBase with positioning function	
05	Waste bottle	500 mL
06	Mini UniPrep Syringeless Filter (LC filter vial)	
07	Filter device	
08	Needle clean device	
09	Injection needle	
10	Reagent bottle rack	

01. Optical table

The size of the optical table is: 600×900 mm, and the M6 threaded holes with a distance of 25 mm on the table are used for positioning and installing other equipment.

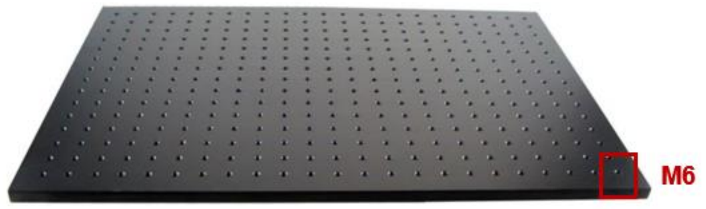

Figure S30. Optical table

02. SCARA (robot) with an electric gripper installed

The model of SCARA is HITBOT Z-Arm 2442, and the model of electric gripper is HITBOT Z-EFG-20. The electric gripper is installed in the corresponding position of the SCARA through the flange plate and screws provided by the manufacturer, and connected to the RS485 interface

of the SCARA to complete the communication connection. The SCARA is connected to the optical platform through a connection board, and the computer network cable port is connected to the SCARA network cable port through the network cable to complete the communication connection.

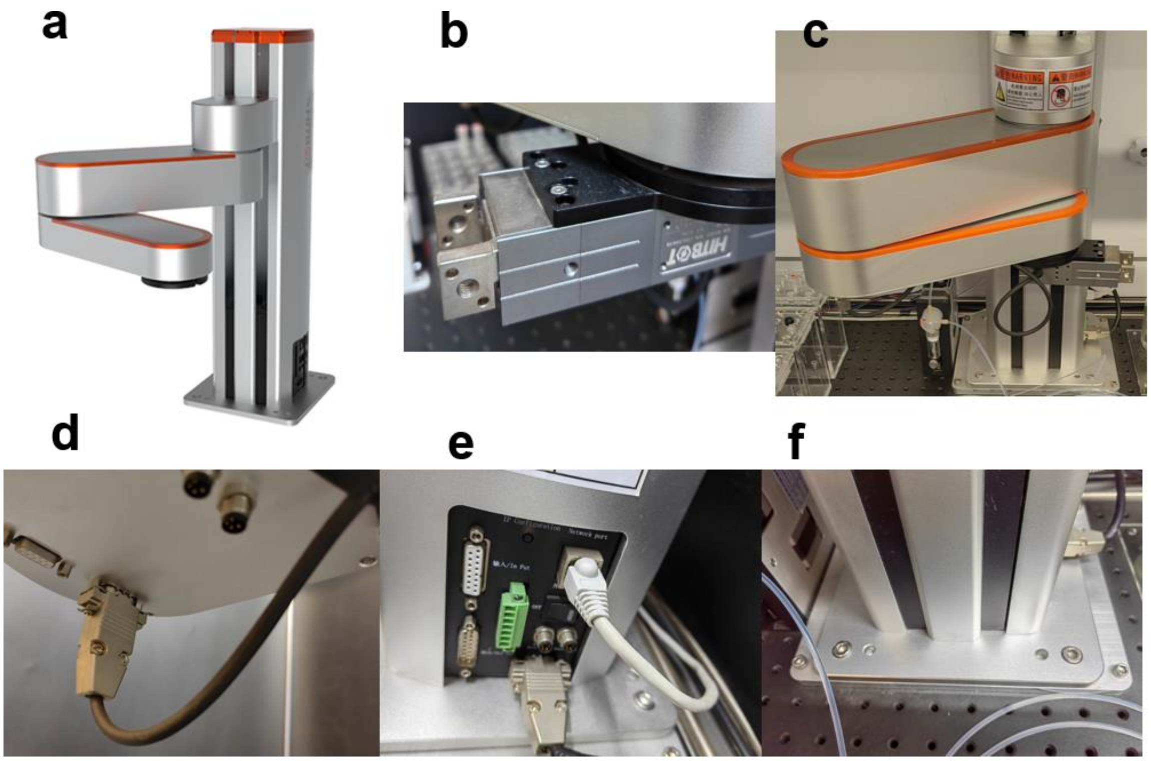

Figure S31. a) SCARA. b) The electric gripper. c) SCARA equipped with the electric gripper. d) The RS485 interface of the SCARA. e) The SCARA network cable port. f) The connection board.

03. Syringe pump

The model of syringe pump is LONGER MSP1-CX. The pump uses a 5 ml glass syringe, and connected to the computer through the RS232/RS485 to USB communication cable to complete the communication. **The E end** of the pump is connected to the cleaning solution bottle filled with acetonitrile, **the O end** of the pump is connected to the needle. All liquid flow paths use FEP tubing and PEEK inverted cone fittings.

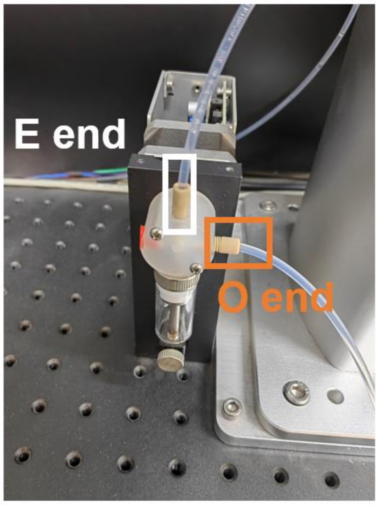

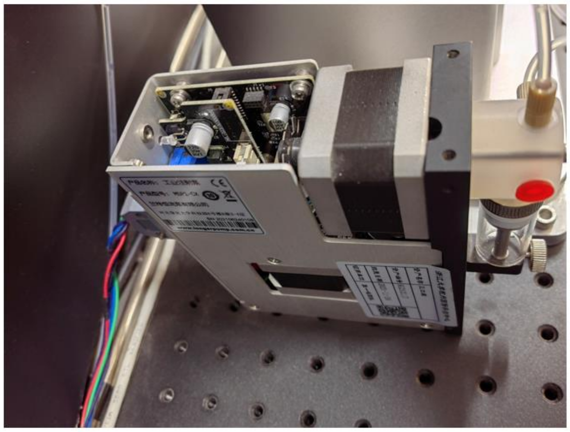

Figure S32. The syringe pump.

04. Wi-eChem Stir & Base with positioning function

On the basis of the above Wi-eChemBase, a photoelectric sensing device is added to realize the positioning function (Figure S33b). This positioning function is mainly used in the following scenarios: when the Wi-eChemStir stops rotating, the photoelectric sensor and the stepping motor can cooperate to realize its rotation to a certain angle, which allows the sampling needle to insert into the bottom of the glass bottle, thereby the liquid in the bottle can be completely withdrawn. Both the signal output end and the power supply end of the photoelectric sensor are connected to the corresponding ports of the stepping motor's driver (Figure S33d).

For Wi-eChemStir, the cathode uses the shape shown in Figure S33a and Figure S33c, so that the needle can insert into the bottom of the glass bottle, thereby the liquid in the bottle can be completely extracted.

Acrylic frame is used to position and fix the Wi-eChemBase (Figure S33a).

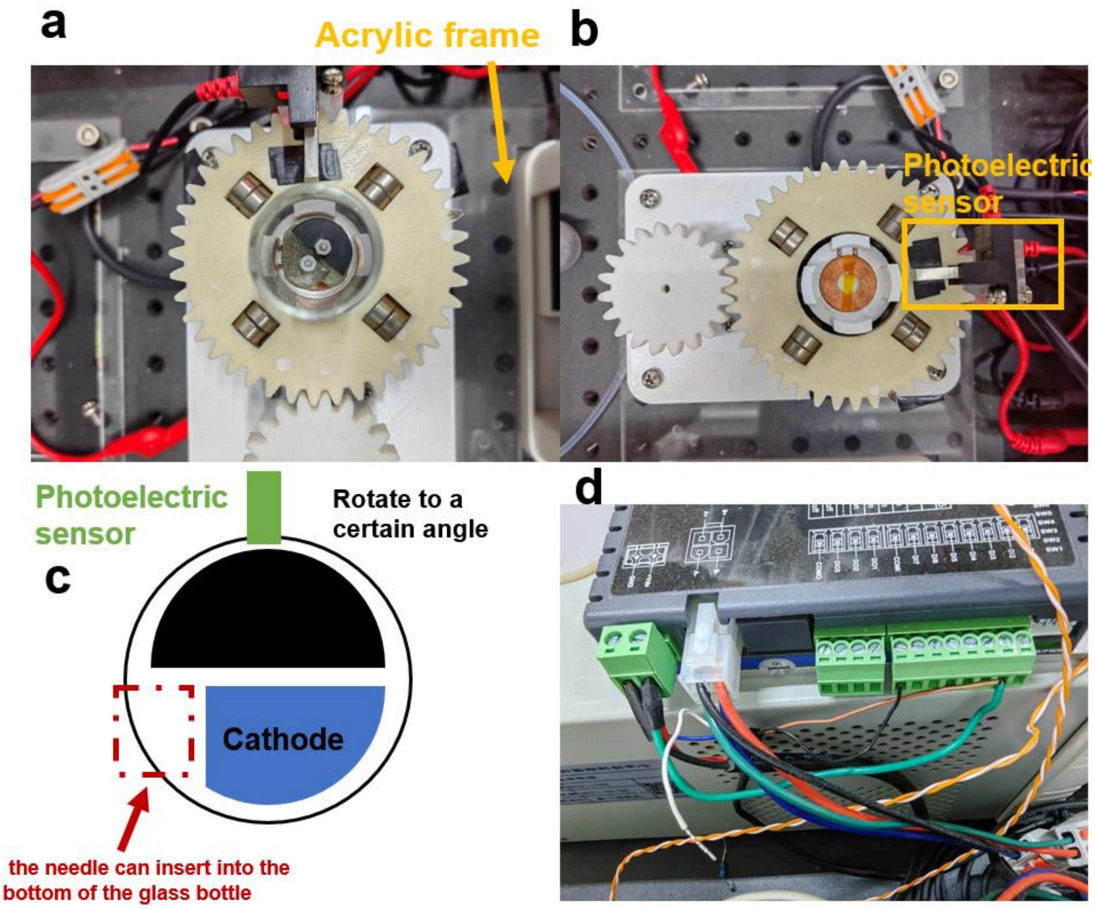

Figure S33. a) b) Wi-eChemStir & Wi-eChemBase with positioning function, c) Electrode diagram, d) The stepping motor's driver

05. Waste bottle

The waste bottle is used to store reaction waste, needle and reactor cleaning solution.

Acrylic frame is used to position and fix the waste bottle.

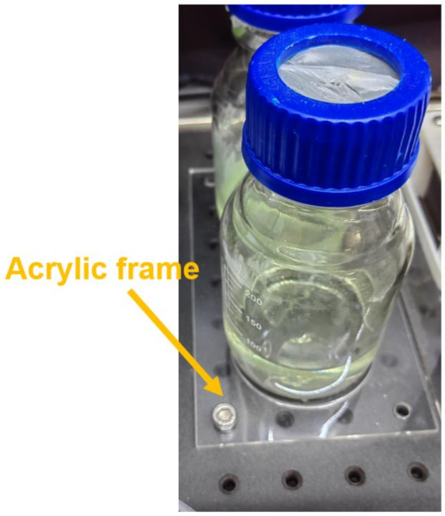

Figure S34. The waste bottle.

06. Mini UniPrep Syringeless Filter 0.45µm PTFE (LC filter vial: chamber and plunger)

The Mini UniPrep Syringeless Filter contains two parts: the outer chamber and the plunger with a filter. The working procedure is as follows: when the plunger is pressed through the sample in the outer chamber (by hand or using a multi-vial compressor) the pressure forces the filtrate through the filter into the reservoir of the plunger. Air escapes through the vent hole until the locking ring is engaged, giving an air tight seal.

The rack for filter vials is fixed directly to the optical platform by means of M6 screws.

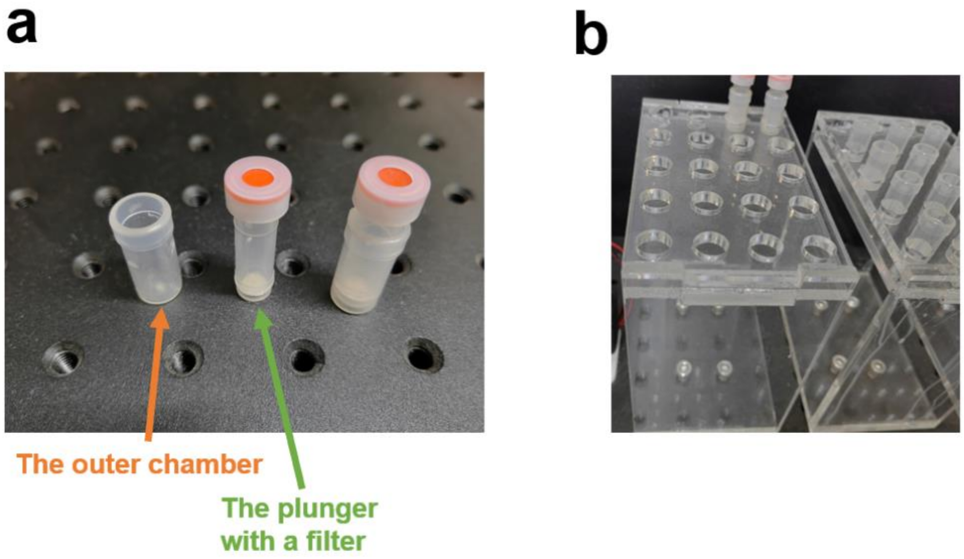

Figure S35 a) The Mini UniPrep Syringeless Filter (LC filter vial), b) Rack for filter vials.

07. Filter device

The filter device is divided into three parts: linear motor, platen, and filter vial placement platform. The linear motor is installed on the side of the filter vial placement platform, while the platen is installed on the linear motor. When the filter device is working, the rod of the linear motor retracts so that the pressure plate is pressed down, thus completing the working procedure of the filter vial (Figure S36a). The assembled CAD design and photo of the filter device is shown in Figure S36b-e. The filter device is fixed directly to the optical platform by means of M6 screws.

The power supply and control of the linear motor are realized through the relay which is connected to the computer through the RS485 communication cable to complete the communication.

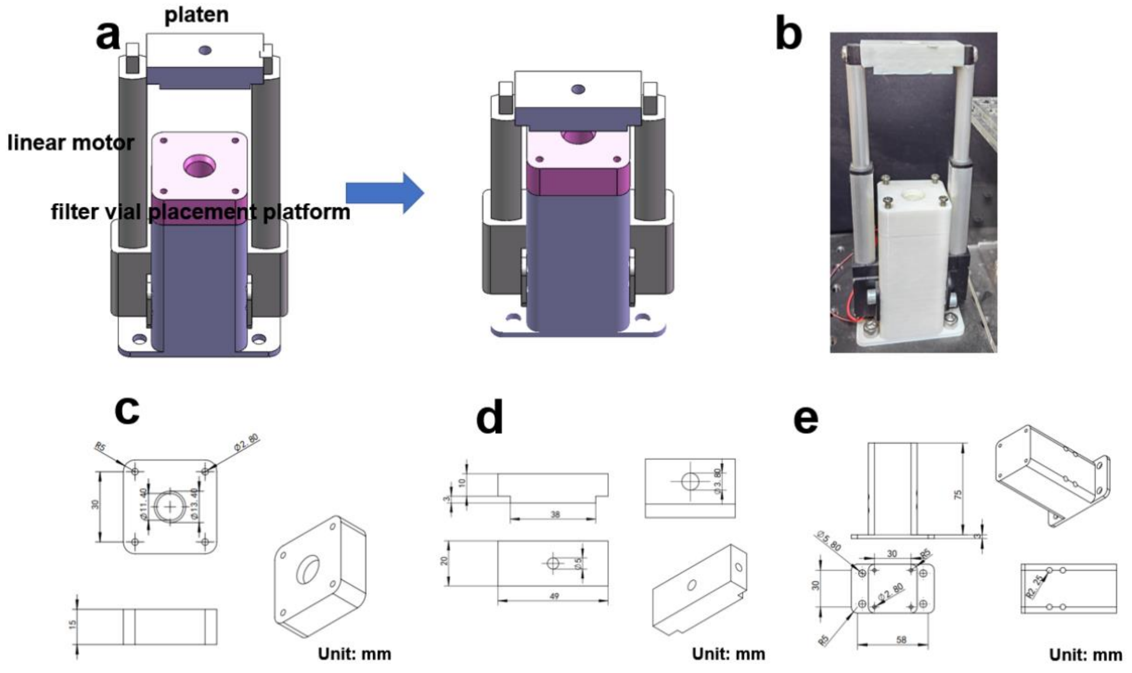

Figure S36. a) Working procedure of the filter device, b) Photo of the filter device, c) d) e) CAD drawings with detailed dimensions.

08. Needle clean device

The needle clean device contains two parts: the peristaltic pump and the washing station. The needle cleaning process is as follows: the injection needle is inserted inside the washing station and the peristaltic pump works to flush the needle surface.

The switch of the peristaltic pump is controlled by the relay. Acrylic frame is used to position and fix the washing station.

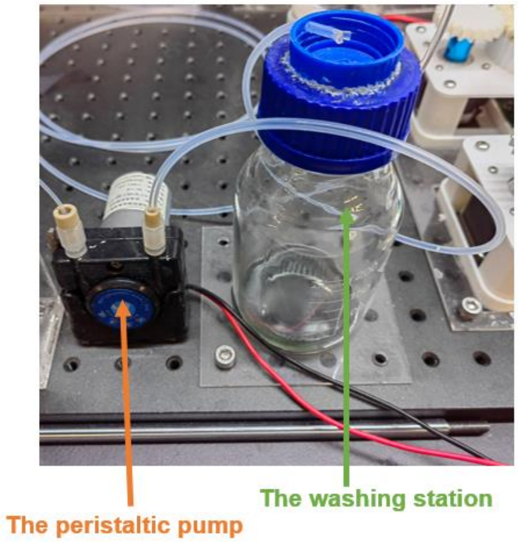

Figure S37. The needle clean device.

09. Injection needle

PEEK luer female thread with 1/4 -28 UNF fitting is used to connect the injection needle and 1/8 FEP tube.

The injection needle is fixed on the needle frame through the needle seat, and the needle seat is taken out from the needle frame by the mechanical gripper when in use, and put back when not in use.

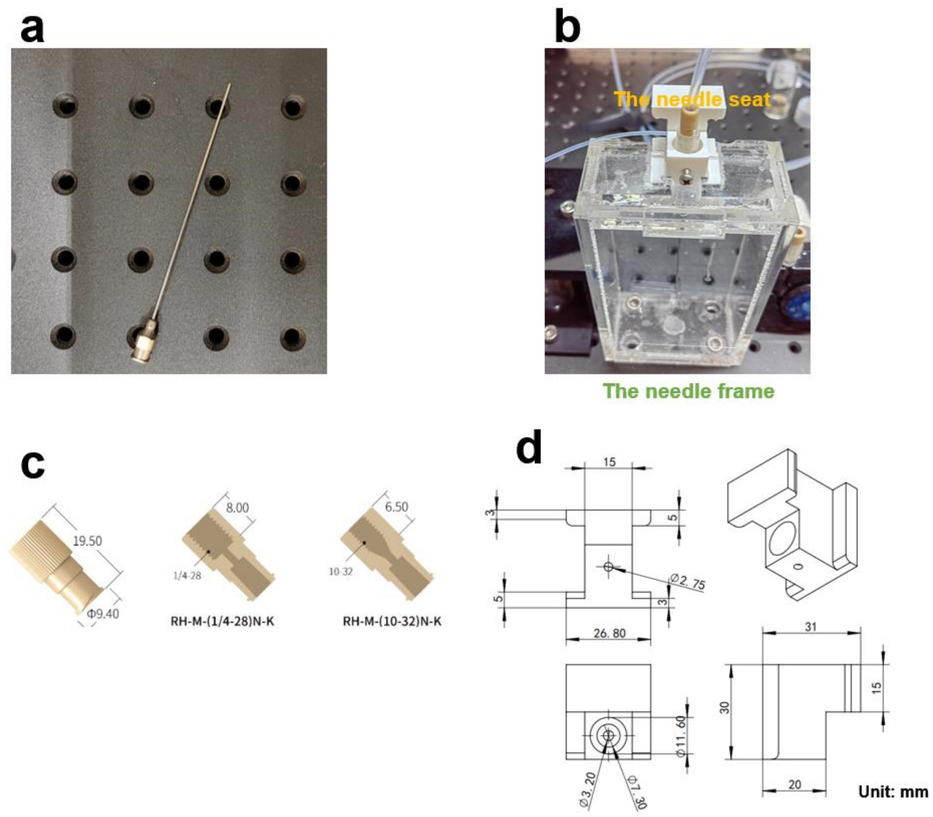

Figure S38. a) The injection needle, b) The needle seat and the needle frame, c) PEEK luer female thread with 1/4 -28 UNF fitting, and d) CAD drawings with detailed dimensions.

10. Reagent bottle rack

The reagent bottle rack has a total of 10 reagent bottles (A-J), which are used to put the reaction solution, cleaning solution and internal standard solution in advance.

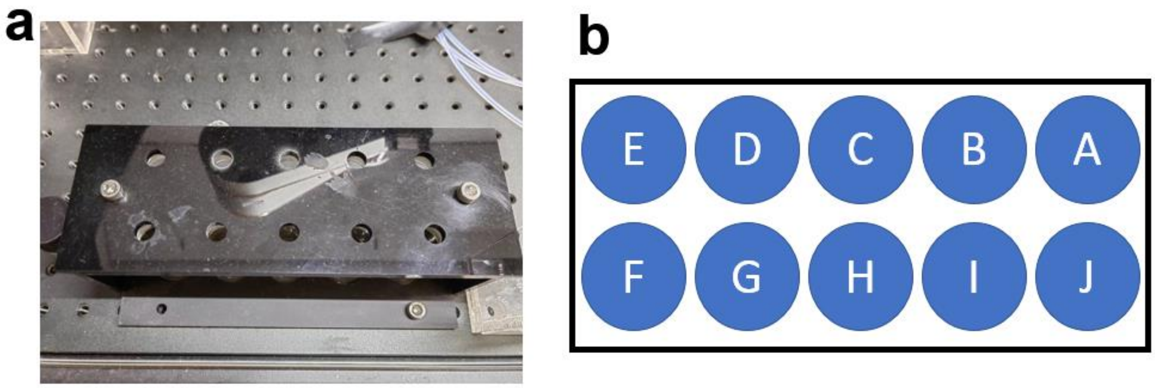

Figure 39. a) The reagent bottle rack. b) The reagent bottle naming system.

12. Nickel catalyzed oxygen atom transfer (OTA) reaction

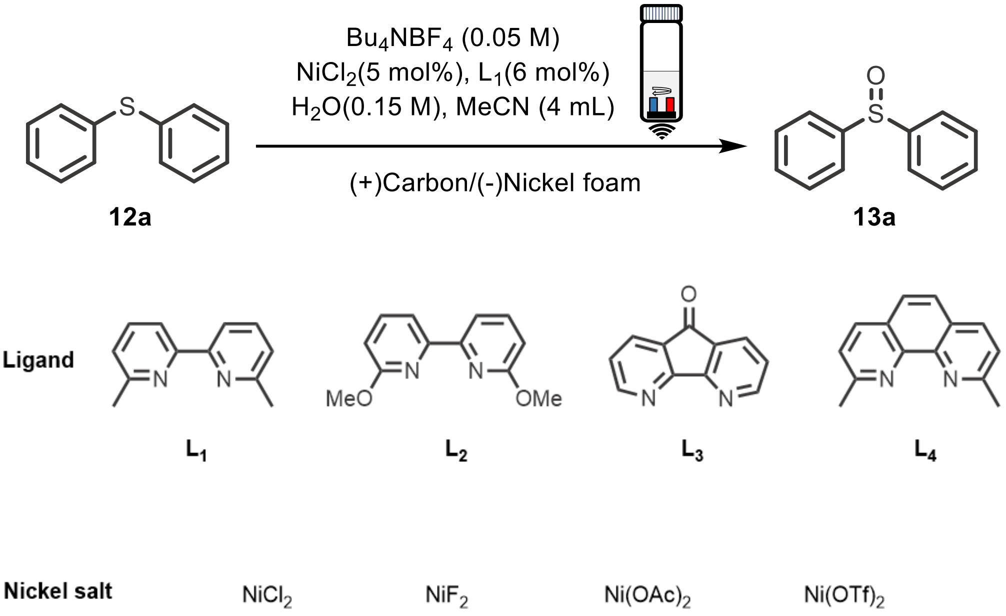

For the nickel catalyzed oxygen atom transfer (OTA) reaction[7], the graphite plate (18 mm diameter semicircle) was used as the anode, the Ni foam was used as the cathode.

Reagent bottle A was charged with diphenyl sulfide (**12a**, 2 mmol), Bu_4NBF_4 (2 mmol) and MeCN (10 mL). Reagent bottle B was charged with nickel salt (5 mol%), ligand (6 mol%) and MeCN (15 mL). Reagent bottle F was charged with internal standard naphthalene (1 mmol) and MeCN (10 mL). Reagent bottle H was charged with HCl (36%-38%, 1 mL) and MeCN (15 mL). The syringe pump was used to withdraw 1 mL and 3 mL of liquid from Regent bottle A and B respectively to form the reaction solution in the preparation stage.

The four glass vials on the Wi-eChemBase were charged with the Wi-eChemStir which equipped with electrodes. The electrolysis voltage was set according to the Excel file (Figure S25). After 1-4 h electrolysis in the reaction stage according to the Excel file. The syringe pump was used to withdraw 0.5 mL of liquid from Reagent bottle F as the internal standard. A sample (0.2 mL) and MeCN (0.2 mL) was taken to the filter vial in the workup stage. The reaction yield was determined by high performance liquid chromatography (HPLC, Agilent 1260). The HPLC calibration curve for determination of yield of target product **13a** is shown in the Figure S40. In the cleaning stage, the four glass vials and Wi-eChemStir equipped with electrodes were washed one time with the cleaning solution withdrawn from Reagent bottle H, and three times with MeCN,

and the syringe pump was used to completely withdraw the liquid in the vial at the end of the cleaning stage. The screening result for four nickel salts is shown in Figure S41.

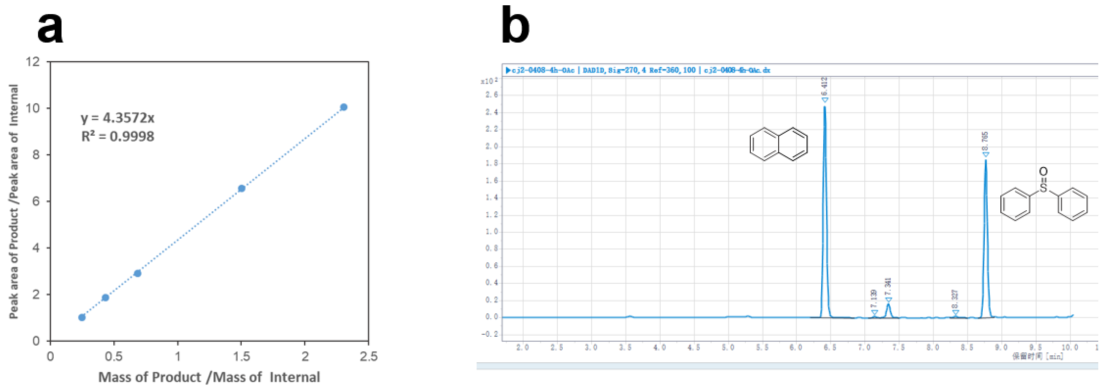

Figure S40. a) HPLC calibration curve for determination of yield of **13a**; b) HPLC chromatograph of **13a** and internal standard.

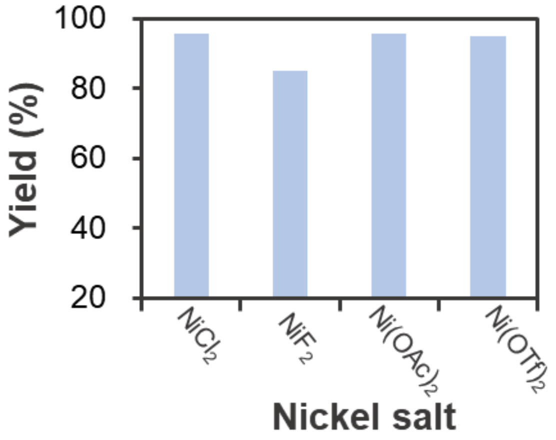

Figure S41. The screening result for four nickel salts.

Substrate screening

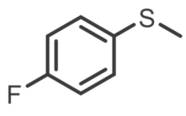
12b

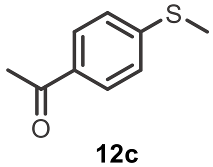
12c

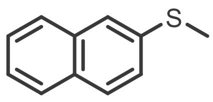
12d

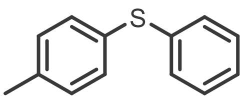
12e

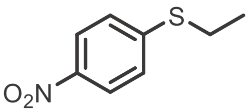
12f

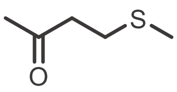
12g

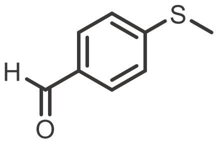
12h

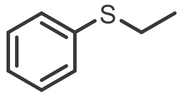
12i

In the substrate screening, Reagent bottle A-D was charged with thioether (**12b**-**12e** in the first- round screening of substrate and **12f-12i** in the second-round screening for substrate, 2 mmol), Bu_4NBF_4 (2 mmol) and MeCN (10 mL). Reagent bottle E was charged with $NiCl_2$ (5 mol%, 6.6 mg), L1 (6 mol%, 11mg) and MeCN (15 mL). Reagent bottle F was charged with internal standard naphthalene (1 mmol) and MeCN (10 mL). Reagent H was charged with HCl (36%-38%, 1 mL) and MeCN (15 mL). The syringe pump was used to withdraw 1 mL and 3 mL of liquid from Regent bottle A-D and E respectively to form the reaction solution in the preparation stage.

The four glass vials on the Wi-eChemBase were charged with the Wi-eChemStir which equipped with electrodes. The electrolysis voltage was set according to the Excel file (Figure S25). After 1-4 h electrolysis in the reaction stage according to the Excel file. Then the reaction solution was removed from the vial, the vial and the Wi-eChemStir were cleaned three times with MeCN, the cleaning solution and reaction solution were combined.

The most of MeCN was removed by rotary evaporation, dibromomethane (0.1 mmol, 7 μL) was added as internal standard. The reaction yield was determined by ^{1}H NMR with the sample diluted by a $CDCl_3$.

13. References

[1] Md. Ruhul Amin, R. B. Roy, in *8th Int. Conf. Softw. Knowl. Inf. Manag. Appl. Ski. 2014*, **2014**, pp. 1–5.

[2] A. J. Bard, L. R. Faulkner, *Electrochemical Methods : Fundamentals and Applications*, New York : Wiley, **2001**.

[3] W. Qi, J. Lai, W. Gao, S. Li, S. Hanif, G. Xu, *Anal. Chem.* **2014**, *86*, 8927–8931.

[4] T. Tajima, H. Kurihara, T. Fuchigami, *J. Am. Chem. Soc.* **2007**, *129*, 6680–6681.

[5] A. N. Kursunlu, E. Guler, H. Dumrul, O. Kocyigit, I. H. Gubbuk, *Appl. Surf. Sci.* **2009**, *255*, 8798–8803.

[6] P. Hu, B. K. Peters, C. A. Malapit, J. C. Vantourout, P. Wang, J. Li, L. Mele, P.-G. Echeverria, S. D. Minteer, P. S. Baran, *J. Am. Chem. Soc.* **2020**, *142*, 20979–20986.

[7] Y. Liang, S.-H. Shi, R. Jin, X. Qiu, J. Wei, H. Tan, X. Jiang, X. Shi, S. Song, N. Jiao, *Nat. Catal.* **2021**, *4*, 116–123.
