# Supplementary material for: Wireless Electrochemical Reactor for Accelerated Exploratory Study of Electroorganic Synthesis
Source: ACS Cent Sci. 2023 Sep 5;9(9):1820–6. doi: 10.1021/acscentsci.3c00856 (PMC10540286; doi:10.1021/acscentsci.3c00856)
Supplement: Supplementary file 4 — oc3c00856_si_004.pdf [file oc3c00856_si_004.pdf]

oc-2023-00856h.R1

Name: Peer Review Information for "Wireless Electrochemical Reactor for Accelerated Exploratory Study of Electroorganic Synthesis"

First Round of Reviewer Comments

Reviewer: 1

Comments to the Author

The authors have proposed and demonstrated a novel concept of wireless electrochemistry (Wi-eChem) based on wireless power transfer technology. The manuscript demonstrates an innovative and versatile system that simplifies the operation of electrochemical reactions, eliminating the need for wire connections. This system's remarkable simplicity allows it to be easily integrated into a fully automated robotic synthesis platform, providing a new avenue for autonomous parallelized electrosynthesis screening. The authors have successfully validated the controllability, reusability, and versatility of the Wi-eChem system with a series of modern electrosynthesis reactions. This includes electrodecarboxylative etherification, electroreductive olefin-ketone coupling, and electrochemical nickel-catalyzed oxygen atom transfer reaction. This technological advancement is hopeful in promoting the further development of organic electrosynthesis. Therefore, I recommend accepting this manuscript for publication, subject to addressing the issue raised.

The setup of the electrodes appears not to be parallel, which may affect the local current density on the electrodes, leading to uneven distribution of current. This could potentially impact reaction development and adoption in a different setting, especially for systems of high current density.

Reviewer: 2

Comments to the Author

I have read the paper Mo and Chen with great interest. I particularly like the concept of Wi-eChem based on wireless power transfer technology as it offers a novel approach to execute electrochemical reactions. One particular interesting feature which the authors did not highlight enough in my opinion is the opportunity to tune the mass transfer by increasing the rpm. This is of high interest to remove any mass transfer limitations. See also this paper: Org. Process Res. Dev. 2021, 25, 7, 1619–1627, which should be cited and the aspect of mass transport should be a bit more detailed.

The dual-function wireless electrochemical magnetic stirrer is a key innovation that provides electrolysis driving force and mixing simultaneously in a miniaturized form factor, demonstrating the system's controllability and reusability. The ability to perform modern electrosynthesis reactions with Wi-eChem, including electrodecarboxylative etherification, electroreductive olefin-ketone coupling, and electrochemical nickel-catalyzed oxygen atom transfer reaction, highlights its versatility.

Moreover, the remarkably simplified operation of Wi-eChem, eliminating the need for wire connections during electrochemical reactions, enables seamless integration into fully automated robotic synthesis platforms for autonomous parallelized electrosynthesis screening. Especially that last platform is for me another reason to support this paper.

I do admit that I thought of the paper as a bit a gimmick for electrochemistry. But the authors did a lot of effort to show its versatility. It remains to be seen if it will be widespread technology, however, I do like the concept and I look forward to seeing follow up work on this topic.

Author's Response to Peer Review Comments:

Please see the attached fille for the response to reviewers's comments.

Reviewer: 1

The authors have proposed and demonstrated a novel concept of wireless electrochemistry (Wi-eChem) based on wireless power transfer technology. The manuscript demonstrates an innovative and versatile system that simplifies the operation of electrochemical reactions, eliminating the need for wire connections. This system's remarkable simplicity allows it to be easily integrated into a fully automated robotic synthesis platform, providing a new avenue for autonomous parallelized electrosynthesis screening. The authors have successfully validated the controllability, reusability, and versatility of the Wi-eChem system with a series of modern electrosynthesis reactions. This includes electrodecarboxylative etherification, electroreductive olefin-ketone coupling, and electrochemical nickel-catalyzed oxygen atom transfer reaction. This technological advancement is hopeful in promoting the further development of organic electrosynthesis. Therefore, I recommend accepting this manuscript for publication, subject to addressing the issue raised.

The setup of the electrodes appears not to be parallel, which may affect the local current density on the electrodes, leading to uneven distribution of current. This could potentially impact reaction development and adoption in a different setting, especially for systems of high current density.

Thank you for the suggestion.

It is true that the non-parallel electrode configuration could potentially lead to uniform current distribution. On one hand, the small size of the miniaturized electrode (18 mm * 9 mm) used in this work. On the other hand, for electrochemical reactions that are sensitive to the current distribution on the electrode, a modified Wi-eChemStir design with parallel electrode setup was also constructed and demonstrated (see Section 3 in Supporting Information).

The parallel electrodes are installed on the original Wi-eChemStir using an electrical conducting adaptor. This new version was validated in the electrodecarboxylative etherification, and the reaction results and device stability were similar to the original Wi-eChemStir (Figure S17).

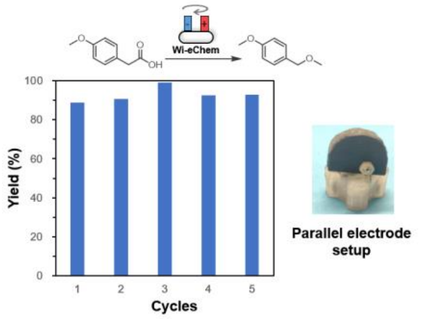

Five repeated experiments conducted with the same Wi-eChemStir (parallel electrode setup) gave stable yields

Reviewer: 2

I have read the paper Mo and Chen with great interest. I particularly like the concept of Wi-eChem based on wireless power transfer technology as it offers a novel approach to execute electrochemical reactions. One particular interesting feature which the authors did not highlight enough in my opinion is the opportunity to tune the mass transfer by increasing the rpm. This is of high interest to remove any mass transfer limitations. See also this paper: Org. Process Res. Dev. 2021, 25, 7, 1619–1627, which should be cited and the aspect of mass transport should be a bit more detailed.

The dual-function wireless electrochemical magnetic stirrer is a key innovation that provides electrolysis driving force and mixing simultaneously in a miniaturized form factor, demonstrating the system's controllability and reusability. The ability to perform modern electrosynthesis reactions with Wi-eChem, including electrodecarboxylative etherification, electroreductive olefin-ketone coupling, and electrochemical nickel-catalyzed oxygen atom transfer reaction, highlights its versatility.

Moreover, the remarkably simplified operation of Wi-eChem, eliminating the need for wire connections during electrochemical reactions, enables seamless integration into fully automated robotic synthesis platforms for autonomous parallelized electrosynthesis screening. Especially that last platform is for me another reason to support this paper.

I do admit that I thought of the paper as a bit a gimmick for electrochemistry. But the authors did a lot of effort to show its versatility. It remains to be seen if it will be widespread technology, however, I do like the concept and I look forward to seeing follow up work on this topic.

Thank you for this suggestion. The suggested reference was properly cited.

We have explored the influence of rotation speed on the electrodecarboxylative etherification under a higher electrolysis potential, such that the increased reaction rate posed a more stringent requirement for mass transfer rate. It was found that a higher rotation speed can suppress the overoxidation of the product through rapid surface renewal of the electrode surface species (see Section 8 in Supporting Information).

In addition, a comparative mass transfer behavior simulation study of Wi-eChem reactor and conventional electrochemical reactor was conducted using the finite element analysis. The conventional electrochemical batch cells with static electrodes and stirred electrolyte typically only have tangential flow velocity across the electrode surface. In comparison, the self-rotating electrodes of Wi-eChemStir can generate a fluid flow that has perpendicular velocity component towards the electrode surface, just like a rotating disk electrode setup (Levich equation). This perpendicular flow pattern can give additional enhancement of the mass transfer, leading to a higher

order dependence of limiting current on the rotation speed. Thus, the mass transfer limiting current of Wi-eChem setup shows a stronger dependence on the stirring speed than that of the conventional electrochemical reactor. The results are added as Section 5 in Supporting Information.
